# Characterization of the Structure and Function of the Photosynthetic RC–LH1 Core Supercomplex From *Rhodospirillum rubrum*

**DOI:** 10.1111/ppl.70275

**Published:** 2025-05-19

**Authors:** Bern Christianson, Zekun Liu, Yingyue Zhang, Chen Wang, Adrian M. Gardner, Yu‐Zhong Zhang, Peng Wang, Lu‐Ning Liu

**Affiliations:** ^1^ Institute of Systems, Molecular and Integrative Biology University of Liverpool Liverpool UK; ^2^ MOE Key Laboratory of Evolution and Marine Biodiversity, Frontiers Science Center for Deep Ocean Multispheres and Earth System & College of Marine Life Sciences Ocean University of China Qingdao China; ^3^ Department of Chemistry, Stephenson Institute of Renewable Energy, and Early Career Laser Laboratory University of Liverpool Liverpool UK; ^4^ Marine Biotechnology Research Center, State Key Laboratory of Microbial Technology Shandong University Qingdao China

**Keywords:** bacterial photosynthesis, electron transport, light‐harvesting, membrane protein, RC–LH1

## Abstract

Photosynthetic reaction center‐light harvesting 1 (RC–LH1) core supercomplexes are essential for energy capture and electron transport in purple bacteria. *Rhodospirillum rubrum* , a model organism for bacterial photosynthesis, features an RC–LH1 architecture with a closed LH1 ring and lacks the peripheral LH2 antenna in the photosynthetic membranes. How this unique RC–LH1 supercomplex performs energy transfer and quinone transport remains unclear. Here, we characterized both the structural and functional properties of *Rsp*. *rubrum* RC–LH1 supercomplex using cryo‐electron microscopy (cryo‐EM), transient absorption (TA) spectroscopy, and cytochrome *c*_2_ oxidation assays. Cryo‐EM of the RC–LH1 monomeric structure revealed a closed LH1 ring of 16 αβ‐polypeptides encircling the RC, with weaker RC–LH1 interactions than other RC–LH1 structures reported. TA spectra and cytochrome *c*_2_ oxidation assays showed that *Rsp*. *rubrum* RC–LH1 monomer with a closed LH1 ring exhibits slower and more distributed excitation energy transfer (EET) kinetics from LH1 to RC and slower electron transport rates than *Rba. sphaeroides* RC–LH1 monomer with a large opening in the LH1 ring. Our findings provide insight into the unique architecture and spectroscopic properties of *Rsp*. *rubrum* RC–LH1 supercomplex. This study enhances our understanding of bacterial photosynthetic mechanisms and lays the foundation for bioengineering applications in artificial photosynthetic systems.

## 1 Introduction

Photosynthetic light‐harvesting (LH) and reaction center (RC) complexes are fundamental components of the photosynthetic apparatus of purple phototrophic bacteria (Swainsbury et al. 2023; Liu et al. 2024). The LH complexes in purple bacteria typically consist of pigment‐protein assemblies that are essential for capturing light energy. There are usually two types of LH antennae complexes: LH1 and LH2. The RC is surrounded by the LH1 complex, forming an RC–LH1 core supercomplex. In some species, LH2 complexes exist as peripheral structures that serve as additional photon absorbers, funnelling energy to the RC–LH1 core. These complexes are specifically arranged in photosynthetic membranes, ensuring efficient energy transduction and electron transfer to cytochrome *bc*_1_ and ATP synthase for ATP generation (Liu and Scheuring 2013; Mullineaux and Liu 2020).

*Rhodospirillum* (*Rsp*.) *rubrum* is an anoxygenic phototrophic bacterium that has been extensively studied as a model organism for bacterial photosynthesis and metabolic processes (Miller et al. 1987; Ghosh et al. 1988; Parkes‐Loach et al. 1988). *Rsp*. *rubrum* exhibits a unique photosynthetic apparatus compared to that of many other purple bacteria. It lacks the peripheral LH2 complex and cytochrome *c* subunit in the RC (Miller et al. 1987; Ghosh et al. 1988; Parkes‐Loach et al. 1988; Fotiadis et al. 2004). This structural simplicity, along with distinctive metabolic characteristics, such as the production of both rhodoquinones (RQ) and ubiquinones as electron carriers (Hiraishi and Hoshino 1984; Imhoff 1984), makes *Rsp*. *rubrum* an attractive model system for elucidating the structural and functional mechanisms of bacterial photosynthesis. Furthermore, the bacteriochlorophyll (BChl) *a* molecules in *Rsp*. *rubrum* are esterified with geranylgeraniol instead of the more common phytol, which further distinguishes its photosynthetic machinery from that of other purple bacteria (Katz et al. 1972; Mizoguchi et al. 2015).

Previous studies have determined the structure of *Rsp*. *rubrum* RC–LH1 supercomplex using two‐dimensional crystallization, single‐particle electron microscopy, atomic force microscopy (AFM), and cryo‐electron microscopy (cryo‐EM) (Ghosh et al. 1993; Karrasch et al. 1995; Jamieson et al. 2002; Qian et al. 2003; Fotiadis et al. 2004). Structural analysis revealed that *Rsp*. *rubrum* RC–LH1 is present as a monomer and possesses a closed, nearly circular LH1 ring structure that encircles the RC. This LH1 ring structure resembled the LH1 structure of *Tch. tepidum* (Yu et al. 2018) but differs from those of other purple bacteria, such as *Rhodobacter (Rba.) sphaeroides, Rba*. *veldkampii*, and *Rba*. *capsulatus*. In the LH1 structures of these species, the presence of a PufX transmembrane polypeptide creates an opening in the LH1 ring, facilitating the rapid diffusion of quinones and quinols across the LH1 ring and efficient electron transport between the RC and cytochrome *bc*_1_ (Bracun et al. 2021, 2023; Qian et al. 2021b; Cao et al. 2022; Tani et al. 2022, 2023; Wang et al. 2024). Other types of gaps have also been observed in the LH1 complexes from *Blastochloris viridis* (Qian et al. 2018) and *Rhodopseudomonas* (*Rps*.) *palustris* (Roszak et al. 2003, Swainsbury et al. 2021). How the closed LH1 architecture of *Rsp*. *rubrum* mediates quinone/quinol transport remains unclear.

Recent findings have also suggested that, within *Rsp*. *rubrum* RC–LH1, there are no intensive interactions between RC and LH1 subunits (Qian et al. 2021a; Tani et al. 2021). It has been proposed that the interactions between RC and LH1 could affect excitation energy transfer (EET) from LH1 to RC by modulating the RC conformation (Thwaites et al. 2023). Therefore, we reasoned that *Rsp*. *rubrum* RC–LH1 may exhibit different EET than other RC–LH1 complexes.

In this study, we investigated extensively the structure, energy, and electron transport of native *Rsp*. *rubrum* RC–LH1 supercomplex using cryo‐EM, transient absorption spectroscopy, and cytochrome *c*_2_ oxidation assays in comparison with the RC–LH1 structures from *Rba. sphaeroides*. Our findings provide insights into the unique architectural and functional features of *Rsp*. *rubrum* RC–LH1 supercomplex.

## 2 Materials and Methods

### 2.1 Growth Condition of *Rsp. rubrum*

Wild‐type *Rhodospirillum* (*Rsp*.) *rubrum* S1 was given by Dr. Daniel Canniffe (University of Liverpool). The *Rsp. rubrum* cells were grown phototrophically under anoxic conditions in liquid M22+ medium (Hunter et al. 1988) supplemented with vitamins (0.08 M nicotinic acid, 0.01 M thiamine, 7.3 mM 4‐aminobenzoic acid, 0.4 mM d‐biotin) at 30°C in sealed glass bottles under a light intensity of 25 μmol photons s^−1^ m^−2^ (Bellight 70 W halogen bulbs).

### 2.2 Thin‐Section Transmission Electron Microscopy

Cells were pelleted by centrifugation (6000 × *g*, 10 min) and processed for thin‐section transmission electron microscopy (TEM) using a Pelco BioWave Pro laboratory microwave system. The cells were first fixed with 0.1 M sodium cacodylate buffer (pH 7.2) supplemented with 2% glutaraldehyde in two steps of 100 W for 1 min each (P1). Samples were then embedded in 4% agarose, followed by staining with 2% osmium tetroxide and 3% potassium ferrocyanide using three steps at 100 W for 20 s each (P2). The reduced osmium stain was then placed in 1% thiocarbohydrazide solution for 10 min. A second osmium stain was applied using P2 with 2% osmium tetroxide. The sample was made electron‐dense by incubating with 2% uranyl acetate at 4°C overnight. Dehydration was performed with a series of increasing alcohol concentrations (30%–100%) before the cells were embedded in the resin medium. Thin sections (70 nm) were cut using a diamond knife, followed by post‐staining with 3% lead citrate. Images were recorded on an FEI 120 kV Tecnai G2 Spirit BioTWIN transmission electron microscope (equipped with a Gatan Rio 16 camera and DigitalMicrograph software).

### 2.3 Purification of RC–LH1 Complexes

Cells were harvested by centrifugation at 5000 × *g* for 10 min at 4°C, washed three times with Tris–HCl (pH 8.0), and resuspended in 20 mM HEPES (pH 8.0). The cells were disrupted by passage through a French press thrice at 16,000 psi. Cell debris was removed by centrifugation at 20,000 × *g* for 30 min. Membranes were collected by centrifuging the resulting supernatant at 125,000 × *g* for 90 min and solubilized by the addition of β‐DDM (n‐dodecyl β‐D‐maltoside) (Melford, Catalog No. D12000) to a final concentration of 3% (w/v) for 30 min to 60 min in the dark at 4°C with gentle stirring. Unsolubilized proteins were removed by centrifugation at 21,000 × *g* for 30 min. The supernatant was then applied onto 10%–25% (w/v) continuous sucrose gradients made with a working buffer containing 0.01% (w/v) β‐DDM. Gradients were centrifuged at 230,000 × *g* for 18 h. The RC–LH1 complexes in the sucrose gradient solution were collected, and the purity of RC–LH1 complexes was characterized by sodium dodecyl sulfate‐polyacrylamide gel electrophoresis (SDS‐PAGE) and absorption spectra (Figure S1).

### 2.4 Absorption Spectra

Purified RC–LH1 complexes were collected from sucrose gradients, and absorbance was measured from 300 to 900 nm at 1‐nm intervals at room temperature using a Libra S22 spectrophotometer (Biochrom).

### 2.5 Transient Absorption (TA) Spectra

TA spectroscopy was conducted using a Harpia‐TA spectrometer (Light Conversion). The Pharos‐SP‐10 W laser system (Light Conversion) produced the probe and pump, operating at 1028 nm with a frequency of 10 kHz and a full width at half maximum (FWHM) of approximately 170 fs. The pump beam was tuned to the desired wavelength using an OPA (Orpheus, Light Conversion) with a beam diameter of ~600 μm (1/e^2^ diameter) at the sample. The pump beam was chopped, resulting in an effective pump rate of 5 kHz. The white light probe was generated by focusing the 1028 nm beam onto a sapphire crystal and focused on a 400 μm beam at the sample. The pump polarization was altered to ensure that the pump and probe beams interacted with the sample at a magic angle of 54.7° to eliminate the effect of anisotropy and rotational diffusion on the spectra. Spectra were recorded using an NMOS detector (S3901, Hamamatsu) after dispersion using a spectrograph (Kymera 193i, Andor), allowing probing within the 530–950 nm range. A pump power of 50 μW (effective pumping rate of 5 kHz) was employed for all the samples, which reduced the Exciton‐Exciton Annihilation (EEA) effects while maintaining a good signal‐to‐noise ratio required for data analysis.

The experiments were performed using a 2‐mm path length quartz cuvette; the solution was left undisturbed for the duration of each experiment (1 h 20 min), and the signal remained stable. Samples were diluted to an OD of ~0.1 in Immobilized Metal Ion Affinity Chromatography (IMAC) buffer containing 50 mM sodium ascorbate, which served as a sacrificial electron donor. The pump wavelength was chosen to match the absorption maximum of the ^LH1^BChl(Q_y_) band observed in the steady‐state UV/Vis spectrum of each RC–LH1 supercomplex. Prior to each experiment, the sample underwent 60 s of pump beam irradiation to guarantee that the RC Q_A_ was photochemically reduced, enabling the investigation of Q_A_ inactivation in the charge‐transfer relaxation process within the RC.

The data were initially processed using CarpetView (Light Conversion) to account for chirp correction (performed using the response from a silicon wafer). The scattered pump signals were detected using the experimental setup. To account for this, a 15 nm range on either side of the pump wavelength was excluded before subsequent analysis. Lifetime Density Analysis was performed using OPTIMUS (Slavov et al. 2015). Only the 750–950 nm spectral region, which contains the dominant TA features, was included in the Lifetime Density Analysis fit, which considerably reduced the computational resources required and prevented data smoothing owing to the introduction of low signal: noise data (as a result of the weak TA features observed < 750 nm). In all cases, the data were fitted by employing three Gaussian coherent artifact signals. Inspection of lifetime density maps resulting from lifetime density analysis is complicated owing to the difficulty in accurately representing the pre‐exponential factor magnitude with contour/color maps. We reduced the three‐dimensional lifetime density map to two‐dimensional kinetic traces by integrating the modulus of the pre‐exponential factor between 750 nm and 950 nm for each lifetime. This generated what we term a “lifetime density kinetic trace” (LDKT) (Figure 5). By calculating the wavelength‐dependent average pre‐exponential factor of lifetimes associated with the dominant band in the LDKT with a peak at ~50 ps, we plot the spectral change associated with this kinetic process in two dimensions, which we term the “lifetime averaged difference spectra”.

Details of the analysis of TA data were provided in Supporting Information.

### 2.6 Cytochrome *c*_2_ Oxidation Assays

Assays were conducted using RC–LH1 complexes at a concentration of 15 nM, incorporating 30 μM reduced horse‐heart cytochrome *c*_2_ (Merck, Catalog No. C2506) and various concentrations of ubiquinone‐2 (UQ_2_) (Merck, Catalog No. C8081), ranging from 0 to 40 μM in HEPES buffer (0.01% β‐DDM, pH 8.0). Three 1‐mL sample replicates at each UQ_2_ concentration were incubated at 4°C in the dark overnight to ensure complete dark adaptation before measurements. Measurements were performed using a Cole‐Parmer SP‐800‐UV spectrophotometer. To exclude the excitation light, the sample and reference detector entrances were equipped with a 550 nm short pass (Thorlabs, FES0550). Absorbance was monitored at 550 nm with a 1‐s interval. Excitation light of 40 μW was provided by an 880 nm Light‐emitting diode (Thorlabs, M880F2) and was delivered at an angle of 90° to the measurement beam. Absorbance was recorded for 1 min before a 5‐min illumination period. To account for the variability of the starting optical density of reduced cytochrome *c*_*2*_ (OD_550_) between samples, we normalized the data to the average OD_550_ during the 1‐min steady‐state period before illumination. Light was turned off for the initial 1 min to ensure stable reduced cytochrome *c*_*2*_ condition. The illumination initiated cytochrome *c*_*2*_ oxidation. Measurement was conducted for 5 min to ensure the cytochrome *c*_2_ oxidation reached steady‐state conditions during illumination. Data were processed by fitting the linear initial rate before the equilibrium phase, from 1 s to 10 s (dependent on UQ_2_ concentration), and averaging the rates of all three samples at each UQ_2_ concentration. Rates were converted into catalytic efficiencies using the concentrations of RC–LH1 dimers and reduced cytochrome *c*_2_ calculated with their respective extinction coefficients, and they were fitted with the Michaelis–Menten model using Origin Pro 2021b (OriginLab).

### 2.7 Cryo‐EM Data Collection

A 4 μL aliquot of RC–LH1 sample was applied to a freshly glow‐discharged holey carbon grid (Quantifoil Au R2/1, 200 mesh) with a continuous carbon support. The grid was blotted for 2 s at 100% humidity and 10°C, with a force level of 0, and immediately plunge‐frozen into liquid ethane cooled by liquid nitrogen using a Vitrobot Mark IV (Thermo Fisher). The grids were then loaded into a 300 kV Titan Krios G3i microscope (Thermo Fisher) equipped with a K3 BioQuantum direct electron detector (Gatan) for data acquisition. A total of 4893 movie stacks were automatically recorded using EPU (Thermo Fisher) (Thompson et al. 2019) at a total dose of 50 e^−^Å^−2^ per stack, with a defocus range of −0.8 to −1.8 μm. Super‐resolution mode was employed at a nominal magnification of ×81,000, corresponding to a pixel size of 0.53 Å, with the energy filter slit set to 20 eV.

### 2.8 Data Processing

Data processing was conducted using cryoSPARC (v4.4.1) (Punjani et al. 2017). Patch motion correction and contrast transfer function (CTF) correction were performed, and micrographs with a resolution worse than 6 Å were discarded. From the remaining 4852 micrographs, 834,944 particles were picked using cryoSPARC's Blob picking algorithm. The particles were extracted and subjected to two rounds of 2D classification, yielding 115,123 particles for further analysis. These particles were used for Ab initio reconstruction into two classes. One “best‐looking class” was chosen for non‐uniform refinement in cryoSPARC (v4.4.1), resulting in 97,584 particles and a resolution of 3.21 Å. Another round of non‐uniform refinement, using the “optimize per‐particle defocus” option, produced a final map with a resolution of 3.02 Å. The resolution was estimated based on the gold‐standard Fourier shell correlation at 0.143.

### 2.9 Model Building and Refinement

The structure of the RC–LH1 monomer from *Rsp*. *rubrum* ATCC 11170 [Protein Data Bank (PDB) ID: 7OY8] was initially docked into the cryo‐EM map of the RC–LH1 complex using UCSF Chimera (v1.17) (Pettersen et al. 2004). The RC–LH1 monomer model was manually refined based on cryo‐EM density using Coot (v 0.9.4) (Emsley et al. 2010), followed by real‐space refinement using Phenix (v1.20.1) (Adams et al. 2010). Figures were generated using UCSF Chimera (v1.17) and ChimeraX (v1.16) (Pettersen et al. 2004; Meng et al. 2023).

## 3 Results and Discussion

### 3.1 *Rsp. rubrum* Photosynthetic Membranes and RC–LH1 Isolation

*Rsp. rubrum* lacks the peripheral LH2 antenna, relying solely on the RC–LH1 supercomplex for energy capture and conversion. To characterize the structure and function of the RC–LH1 supercomplex, we grew *Rsp*. *rubrum* phototrophically under anoxic conditions and isolated RC–LH1 supercomplexes using sucrose gradient ultracentrifugation (Figure S1). Thin‐section EM showed that the intracytoplasmic membranes (ICMs) of *Rsp. rubrum* form small membrane vesicles in the cytoplasm (Figure S2A), in agreement with previous observations (Cohen‐Bazire and Kunisawa 1963; Boatman 1964; Scheuring and Sturgis 2009). The apparent diameter of *Rsp*. *rubrum* ICM vesicles (80 ± 11 nm) was greater than those of both *Rba. sphaeroides* WT (57 ± 8 nm) and *ΔpufX* (71 ± 10 nm) ICMs (Figure S2B). Moreover, the vesicular structures of *Rsp*. *rubrum* ICMs appeared relatively irregular compared to those in *Rba. sphaeroides* WT and *ΔpufX* (Figure S2A), *Rba. veldkampii* (Bracun et al. 2021), and *Rba. capsulatus* (Bracun et al. 2023), presumably owing to the lack of LH2 in ICMs. Previous studies have indicated that the *Rsp*. *rubrum* ICMs varied under different cultivation conditions; they were smaller under chemotrophic conditions than under phototrophic conditions (Golecki et al. 1980). Absorption spectra indicated that *Rsp*. *rubrum* RC–LH1 possesses different carotenoid pigments than *Rba. sphaeroides* RC–LH1 (Figure S1D). The structural integrity of the isolated RC–LH1 supercomplexes was verified using sodium dodecyl‐polyacrylamide gel electrophoresis (SDS‐PAGE) (Figure S1D).

### 3.2 Overall Structure of RC–LH1

**FIGURE 1:**
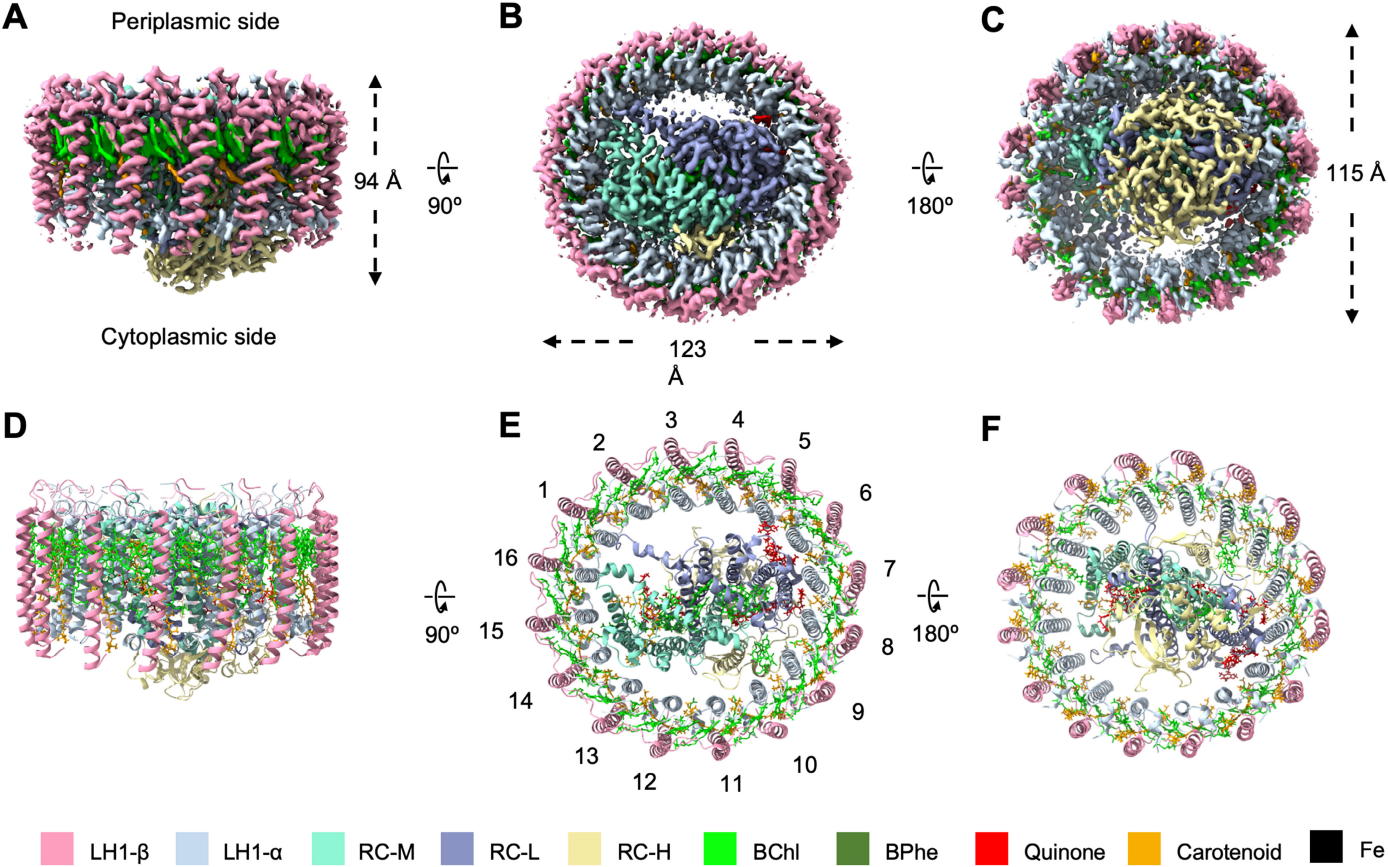
Cryo‐EM structure of the RC–LH1 core complex from *Rsp. rubrum*. (A–C) Views of the RC–LH1 density map, colored as in the key at the bottom. (A) Side view of the RC–LH1 complex in the membrane plane, showing the height of the RC–LH1 complex. (B) Top view of the RC–LH1 complex from the periplasmic side, showing the diameters of the long axes. (C) Bottom view of the RC–LH1 complex from the cytoplasmic side, showing the diameters of the short axes. (D–F) Architectural model of the RC–LH1 complex in three different views corresponding to (A–C). The LH1 subunits are numbered in (E).

**FIGURE 2:**
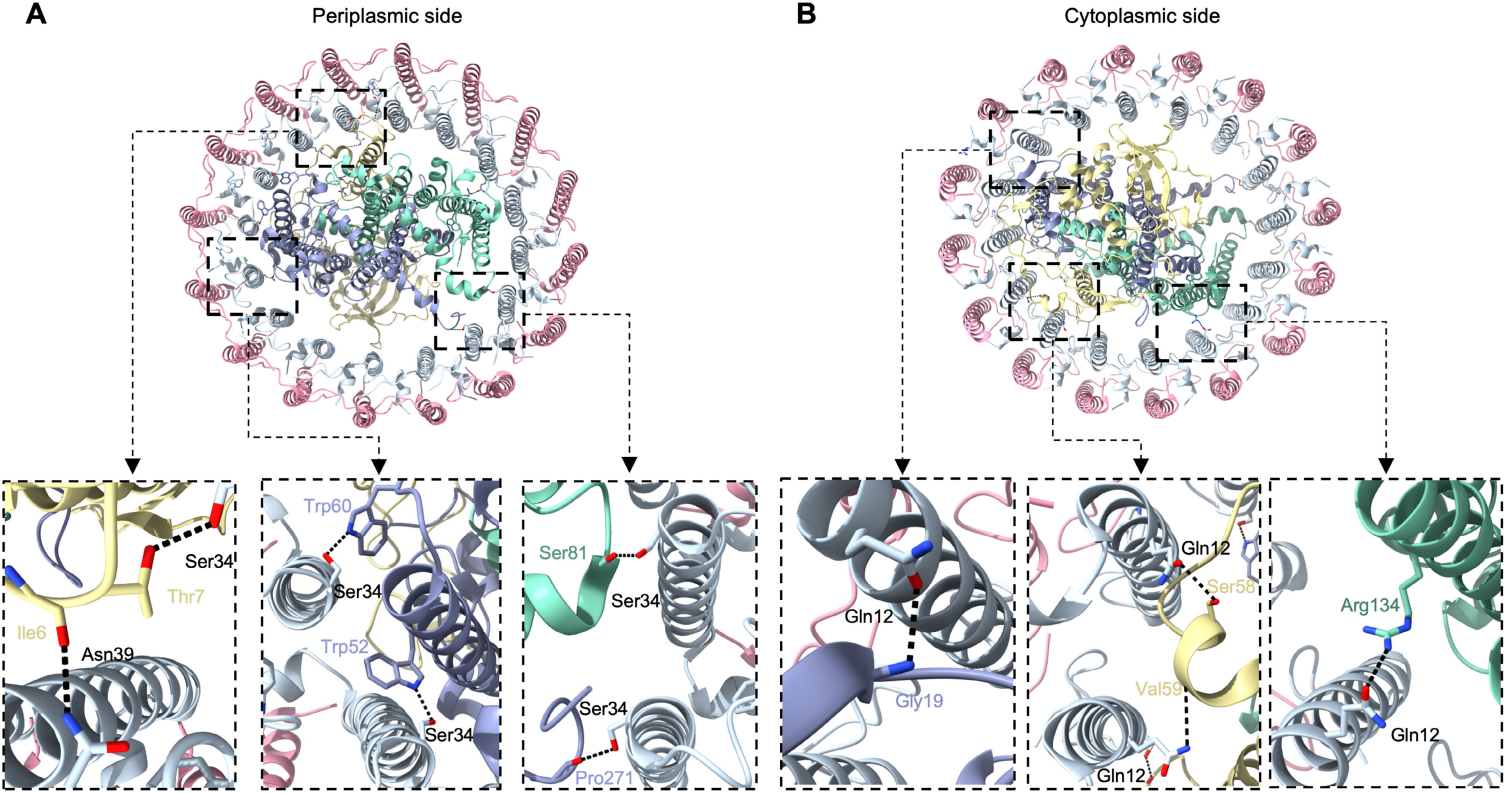
Interaction sites between the RC and LH1 at the periplasmic side (A) and the cytoplasmic side (B). Details are displayed by zoomed‐in views showing residue electron densities and interactions. Distances between interacting residues are shown in Table S2.

The structure of isolated RC–LH1 supercomplexes was characterized by cryo‐EM. A total of 4893 cryo‐EM movies were recorded, of which 834,944 particles were picked for further data processing and 115,123 good particles were used for final 3D reconstruction. The cryo‐EM structure of *Rsp*. *rubrum* RC–LH1 complex was solved at a 3.0 Å resolution (Figure S3). Most amino acid side chains were clearly delineated in the cryo‐EM map (Figure 1A–C), enabling the generation and refinement of the atomic model of the RC–LH1 complex (Figure 2D–F). Statistics for the final model are provided in Table S1.

The cryo‐EM structure of isolated RC–LH1 was largely similar to previously reported structures (Qian et al. 2021a; Tani et al. 2021). The overall shape of the complex is slightly elliptical in projection, with short and long axes of 115 and 123 Å (Figure 1B,C) and 94 Å in height (Figure 1A). When observed perpendicular to the membrane plane, the LH1 complex forms a closed ring comprising 16 pairs of αβ‐polypeptides, 32 BChls, and 16 carotenoids (Figure 1D). The modeled LH1 α‐ and β‐polypeptides of *Rsp*. *rubrum* consist of 45 and 44 amino acid residues, respectively. The α‐ and β‐polypeptides feature a central α‐helical domain flanked by the N‐ and C‐terminal regions, situated close to the cytoplasmic or periplasmic membrane surface, respectively (Figure 1D). *Rsp*. *rubrum* exhibits a distinctive feature in its BChls, with a propionic acid side chain esterified by geranylgeraniol (BChl *a*_GG_) rather than phytol (Francke and Amesz 1995; Qian et al. 2021a; Tani et al. 2021). The central RC complex consists of three protein subunits (H, L, and M), 5 BChls *a*_*GG*_, 2 BPhes, one carotenoid, and 3 quinones (Q_A_, Q_B_, Q_3_).

### 3.3 Interactions Between RC and LH1

LH1 interfaces with RC via hydrogen bonds in six regions on both the periplasmic and cytoplasmic sides (Figure 2; Table S2). On the periplasmic side, H‐Thr7, L‐Trp60, L‐Trp52, L‐Pro271, and M‐Ser81 interact with Ser34 residues of neighboring LH1 α‐subunits. In addition to these conserved hydrogen bond positions in multiple purple bacteria such as *Rba. veldkampii* and *Rba. sphaeroides*, H‐Ile6 in *Rsp*. *rubrum* RC–LH1 complex also interacts with the Asn39 residues of the LH1 α‐subunits. On the cytoplasmic side, the Gln12 residues of the α‐subunits interact with L‐Gly19, H‐Ser58, H‐Val59, and M‐Arg134, which play major roles in interacting with the RC subunits. Interestingly, structural analysis revealed that *Rsp*. *rubrum* RC–LH1 has relatively weaker interactions between RC and LH1 compared to other RC–LH1 complexes (Qian et al. 2018, 2022; Yu et al. 2018; Bracun et al. 2021; Tani et al. 2021; Swainsbury et al. 2023; Wang et al. 2024), which is consistent with recent cryo‐EM structural analysis (Qian et al. 2021a; Tani et al. 2021).

### 3.4 The LH1 Architecture

The LH1 complex of *Rsp*. *rubrum* consists of tightly organized αβ subunits that form the structural framework of the LH1 ring, without additional polypeptides such as PufX identified in *Rhodobacter* species or protein W in *Rps*. *palustris*, which interrupted the formation of a complete LH1 circle. There is one carotenoid per LH1 αβ heterodimer, similar to that in some purple photosynthetic bacteria (Qian et al. 2018, 2022, Yu et al. 2018, Bracun et al. 2021, Tani et al. 2021, Swainsbury et al. 2023).

**FIGURE 3:**
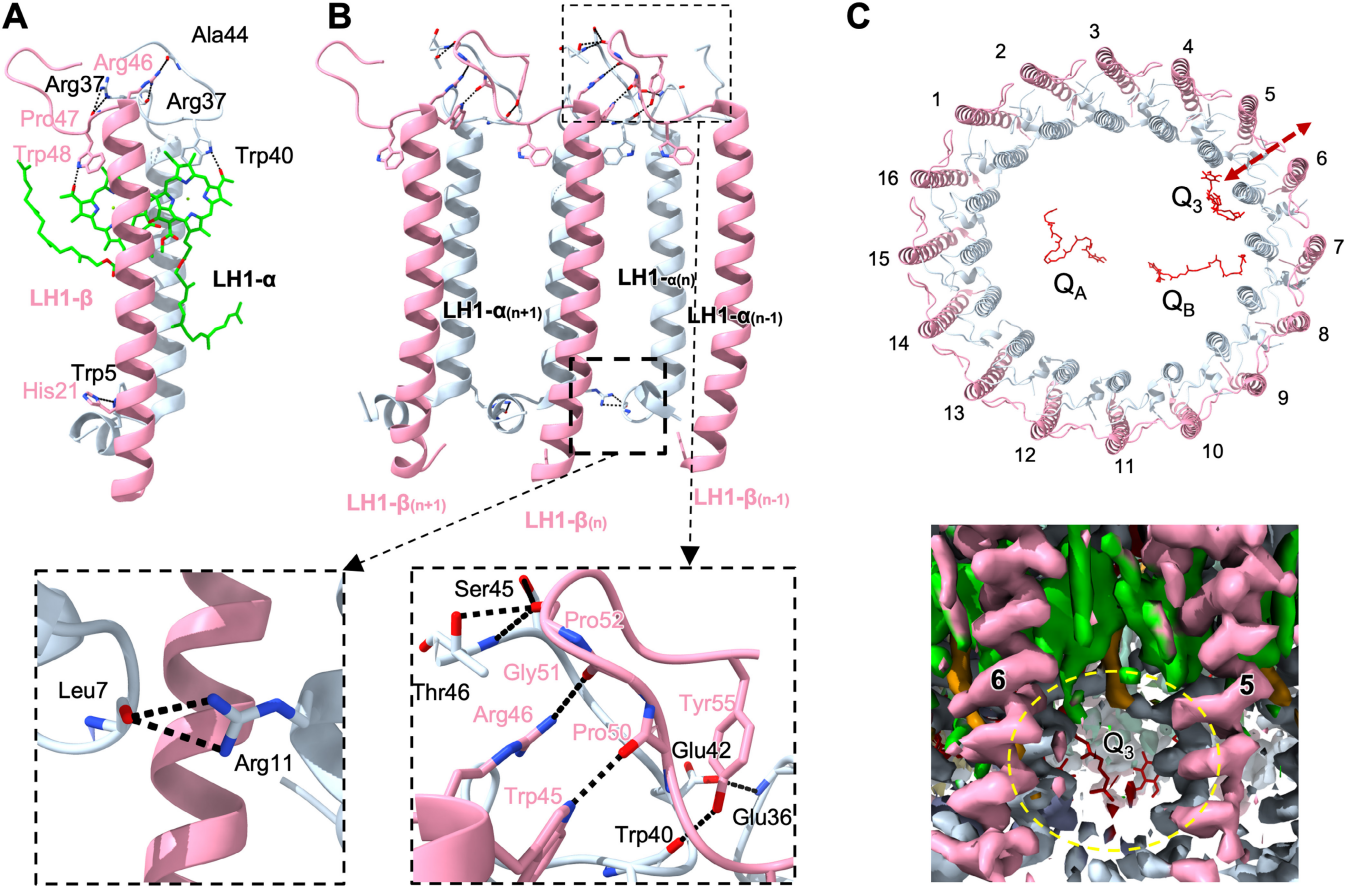
Protein–protein and protein‐pigment interactions within the LH1 association. (A) Interactions within an LH1 subunit. (B) Intra‐ and inter‐subunit interactions within three LH1 αβ‐subunits. Details are displayed by zoomed‐in views showing residue electron densities and interactions. (C) Proposed path taken by a quinol diffusion through the pore (Top). View of the LH1 complex from outside the membrane highlighting the pore location (Bottom). The LH1 α‐polypeptide is light blue sky, the β‐polypeptide is pink, spirilloxanthin is green. Yellow dashed circle highlights the pore. Q_3_ quinone (red) in the background. Distances between interacting residues are shown in Table S3.

The LH1 structure was stabilized by an intricate network of hydrogen bonds (Figure 3A,B). All intra‐heterodimer bonds occur within the N‐ and C‐terminal regions of individual LH1 αβ polypeptide subunits. In the N‐terminal region, β‐His21 forms a hydrogen bond with α‐Trp5. In the C‐terminal region, hydrogen bonds are formed between α‐Arg37 and β‐Pro47, α‐Arg37 and β‐Arg46, and α‐Ala44 and β‐Arg46 (Figure 3B). No hydrogen bonds were identified between the transmembrane segments of the LH1 αβ‐polypeptides. The keto‐oxygens of BChl *a*_GG_ form hydrogen bonds with both β‐Trp48 and α‐Trp40. Additionally, the Mg atoms of BChls *a*_*GG*_ are coordinated by their proximate His residues. These intricate interactions ensure strong associations between the αβ‐polypeptides and pigments.

Figure 3B shows the intermolecular interactions among the LH1 αβ‐polypeptides within *Rsp*. *rubrum* LH1 complex. Extensive inter‐subunit hydrogen bonds between the LH1 αβ‐polypeptides were also formed in the N‐terminal and C‐terminal regions. On the cytoplasmic side, α(n)‐Arg11 forms hydrogen bonds with neighboring α(n‐1)‐Leu7. On the periplasmic side, β(n)‐Trp45, β(n)‐Arg46, α(n)‐Thr46, α(n)‐Ser45, α(n)‐Trp40, and α(n)‐Glu42 formed hydrogen bonds with β(n‐1)‐Pro50, β(n‐1)‐Gly51, β(n‐1)‐Pro52, β(n‐1)‐Pro52, β(n‐1)‐Tyr55, and α(n‐1)‐Glu36, respectively.

Closer inspection revealed a small pore formed by the hydrophobic residues of LH1‐5 and LH1‐6 αβ‐polypeptides in proximity to the quinone molecule Q_3_ (Figure 3C), in agreement with the findings from other *Rsp*. *rubrum* RC–LH1 structures (Qian et al. 2021a, Tani et al. 2021). This pore likely serves as a channel for quinone transport into and out of RC–LH1. Similar paths have also been observed in other RC–LH1 structures (Yu et al. 2018; Bracun et al. 2021, 2023; Swainsbury et al. 2021) and have been suggested by computational simulations (Aird et al. 2007; Mao et al. 2024).

### 3.5 Cofactors in RC–LH1

**FIGURE 4:**
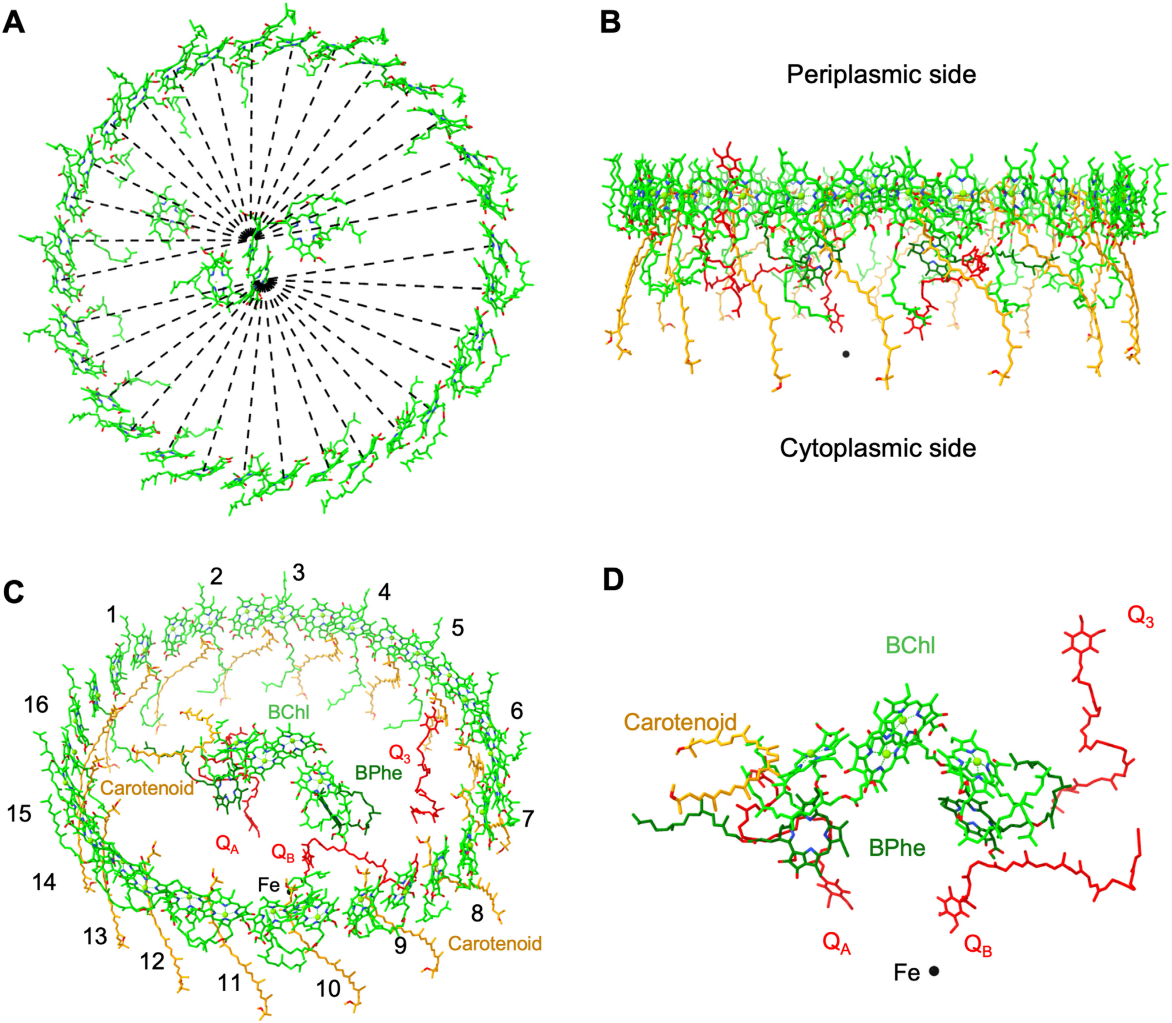
Arrangement of the pigments and cofactors in the RC–LH1 supercomplex. (A) Mg‐Mg distances of BChls in the LH1 ring to the special pair BChls are depicted on a periplasmic view. The distances of these pathways are shown in Table S5. (B) Side view of the architecture of pigments and cofactors in the membrane plane with the periplasm above and the cytoplasm below. (C) Pigments and cofactors viewed from the periplasmic side by tilting 40°. LH1 antenna numbers are marked. (D) Arrangement of the pigments and quinones associated with the RC.

The pigments form a tightly stacked array and are systematically organized into two distinct groups: a ring consisting of 32 closely coupled BChls *a*_GG_ and a ring comprising 16 carotenoids (Figure 4A,B). Due to the elliptical shape of the LH1 ring, the distances between BChls in LH1 vary from 10.19 Å to 7.86 Å (Table S4). Both the Mg‐Mg distances between BChls in individual LH1 αβ‐heterodimers (9.50 Å on average) and between BChls of adjacent LH1 αβ‐subunits (8.70 Å on average) (Figure S5) are within 10 Å, which is critical for efficient exciton coupling and energy resonance within LH1 (Freer et al. 1996; Ye et al. 2012; Bracun et al. 2021). The average Mg‐Mg intra‐subunit distance (9.53 Å) is close to that of RC–LH1 from *Rfl. castenholzii* (9.5 Å) but is greater than that of the RC–LH1 complexes from *Tch. tepidum* (8.88 Å), *Trv*. *strain* 970 (9.01 Å) and shorter than those of the RC–LH1 complexes from *Rps*. *palustris* (9.53 Å) and *Rba. blasticus* (9.9 Å). The distances between BChls in the LH1 ring and the special pair of BChls in the RC vary between 48.43 Å and 37.59 Å (Figure 4A; Table S5), which is important for efficient EET from the LH1 ring to RC.

**FIGURE 5:**
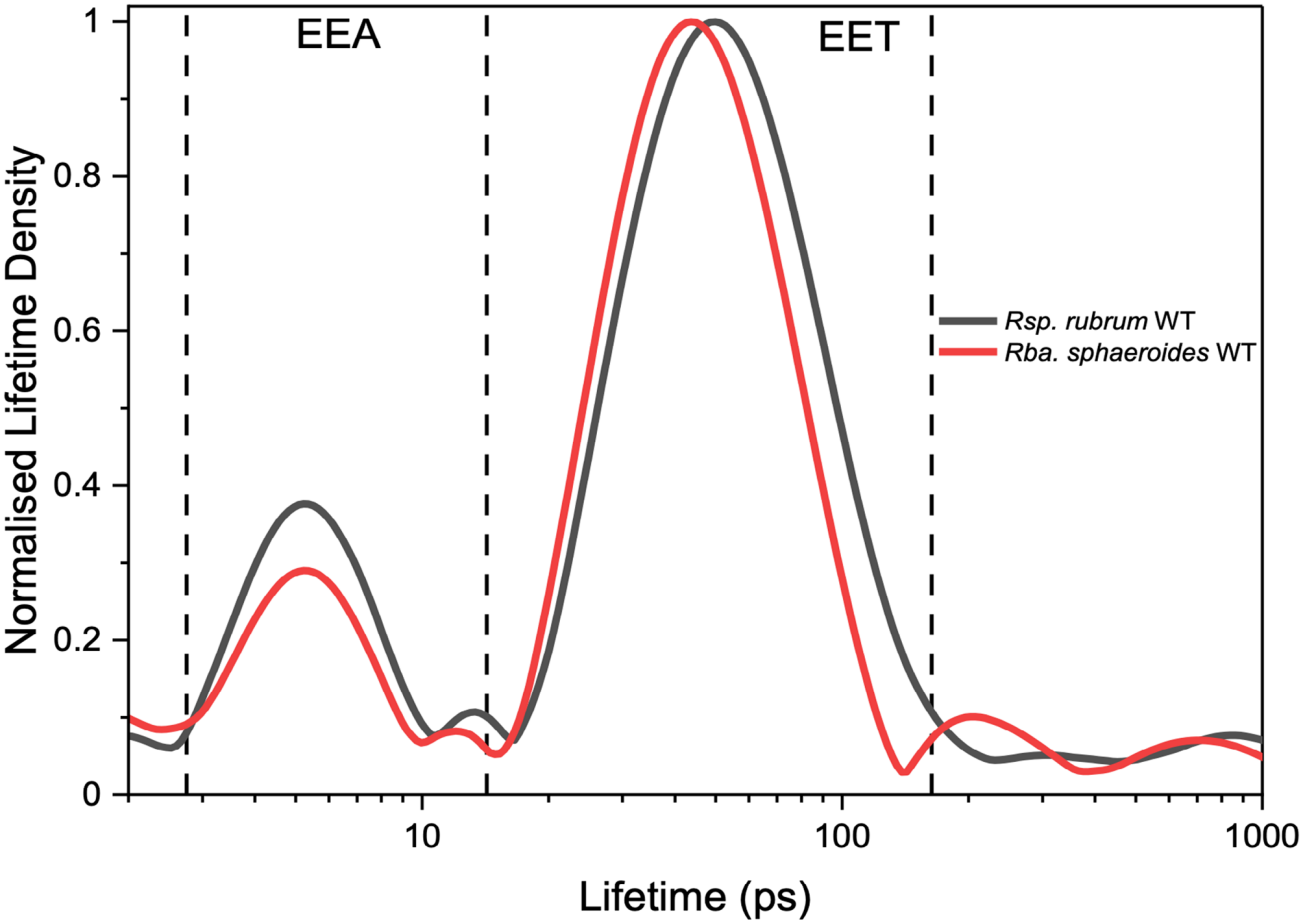
Lifetime density kinetic traces of the RC–LH1 monomers from *Rsp. rubrum* WT and *Rba. sphaeroides* WT, measured by transient absorption (TA) spectroscopy. See also Figure S7.

The cofactors in the RC included five BChls, two BPhes, one carotenoid, one Fe, and three quinones (Figure 4C,D). Unlike the RCs from other purple bacteria, an additional BChl was identified in *Rsp. rubrum* RC. This extra BChl molecule is positioned between the RC‐H and LH1‐α subunits, as previously reported (Qian et al. 2021a). From an energetic perspective, transferring energy from the excited state of LH1 (LH1*) BChls to this extra BChl would be unfavorable because it requires an “uphill” energy transfer. In contrast, transferring energy from this BChl to the RC would be energetically favorable, suggesting it may play a role in energy transfer to the RC. However, neither our TAS results (Figures 5 and S9) nor previous studies employing picosecond TAS (Visscher et al. 1989) identified any distinctions between *Rsp*. *rubrum* and *Rba. sphaeroides* RC–LH1 supercomplexes. While the exact function of this BChl remains to be fully elucidated, its occurrence and unique position emphasize the modular design and the structural and functional flexibility of natural photosynthetic RC‐LH1 supercomplexes (Liu et al. 2024). This variability may confer evolutionary advantages to photosynthetic bacteria, potentially boosting their ability to adapt to various environmental challenges.

### 3.6 Energy Transfer Within RC–LH1

Our structural analysis indicated that weaker interactions are present in the RC–LH1 monomer of *Rsp*. *rubrum* (Figure 2; Table S2), compared to that of *Rba*. s*phaeroides* (Cao et al. 2022). This may be expected to increase the LH1 → RC EET lifetime (Thwaites et al. 2023; Wang et al. 2024). Moreover, the presence of the additional BChl between RC‐H and LH1‐α has been previously hypothesized to expedite EET (Qian et al. 2021a). To explore these possibilities, we examined the picosecond‐nanosecond (ps‐ns) photodynamics of isolated *Rsp*. *rubrum* RC–LH1 supercomplexes compared to the RC–LH1 monomers from *Rba. sphaeroides* WT and Δ*pufX* using transient absorption (TA) spectroscopy (Figures 5, S7 and S8).

To analyze the kinetics of the TA spectra of the RC–LH1 complexes, we applied lifetime distribution analysis to generate three‐dimensional lifetime density maps (Figure S8B). The lifetime density map was then converted into two‐dimensional lifetime density kinetic traces (LDKT) by integrating the modulus of the pre‐exponential factor between 750 and 950 nm for each lifetime to facilitate comparisons between samples, as reported in our previous studies (Thwaites et al. 2023, Wang et al. 2024). By averaging the initial decay rates from LDKT based on the wavelength, we generated two‐dimensional plots, namely lifetime‐averaged difference spectra (LADS), to show the spectral changes for each kinetic process (Figure S8C).

From these LDKTs, the LH1 → RC EET peak lifetimes (τ_EET_) of 50 ps for *Rsp*. *rubrum* WT and 44 ps for *Rba. sphaeroides* WT were obtained (Figure 5). As shown in previous studies (Swainsbury et al. 2021; Thwaites et al. 2023), there is no correlation between the average ^LH1^BChl–^RC^P distances and LH1 → RC EET lifetime. In contrast to the hypothetical role of the additional BChl between the RC‐H and LH1‐α subunits, as revealed by cryo‐EM data, no acceleration of LH1 → RC EET was determined in *Rsp*. *rubrum* WT. The Qy band of *Rsp*. *rubrum* is notably narrower than *Rba. sphaeroides* (Figure S1D), indicating decreased inhomogeneous broadening within the former. An elegant study explored the role of ^LH1^BChl and ^RC^P energy differences, LH1 disorder, and exciton delocalization in determining LH1 → RC EET lifetimes in *Thermochromatium tepidum* RC–LH1 complexes (Ma et al. 2016). Therein, the decreased ordering within the LH1 ring resulted in shorter exciton delocalization lengths. Previous studies have demonstrated that the ratio of photoinduced absorption (PIA) to ground‐state bleach (GSB) can provide insight into the degree of exciton delocalization (Pullerits et al. 1996; Ma et al. 2016). Our analysis reveals striking similarities in this ratio for both *Rsp*. *rubrum* and *Rba. sphaeroides* RC–LH1 monomers (Figure S9), in contrast to the significant differences observed for the ion‐modified *Tch. tepidum* RC–LH1 complexes (Ma et al. 2016). These findings suggest that (on average) the exciton is delocalized across a similar number of chromophores in both *Rsp. rubrum and Rba. sphaeroides*, consistent with the observed similarities in average inter‐ and intra‐subunit ^LH1^BChl distances of RC–LH1 complexes of these species (Table S6) as well as their comparable Qy absorption maximum (Figure S1D). Minor differences in the PIA peak position were observed between the two WT RC–LH1 complexes (Figure S9). However, a direct comparison is challenging due to the overlap of this feature with the larger GSB signal. This overlap perturbs the PIA position and shape, owing to the differences in the UV/Vis absorption (Figure S1D). The comparable degree of excitonic delocalization is supported by the similarity of the shape of the TA negative feature observed at wavelengths beyond ~885 nm, assigned to the overlapping GSB and stimulated emission of ^LH1^BChl, where the shape of the Qy absorption band of both species is remarkably similar. Hence, we found no substantial evidence to suggest significant differences in the (relaxed) excitonic state between the two WT RC–LH1 complexes.

Notably, *Rsp*. *rubrum* WT RC–LH1 monomers exhibited not only a longer τ_EET_ but also a broader distribution compared to *Rba. sphaeroides* WT monomers. We propose that both features result from the weaker interactions between LH1 and RC in *Rsp*. *rubrum* WT RC–LH1, as confirmed by the cryo‐EM results from us and others (Figure 2; Table S2) (Qian et al. 2021a; Tani et al. 2021). The weaker interactions likely result in a less‐constrained orientation of the RC within the LH1 ring. These observations align well with our previous findings, which indicated that the distribution of τ_EET_ observed in LDKT is more strongly influenced by variation in the RC orientation within the LH1 ring than by the ^LH1^BChl–^RC^P physical distances (Thwaites et al. 2023) or by inhomogeneity within the LH1 ring itself.

### 3.7 Electron Transfer of *Rsp. rubrum* RC–LH1

**FIGURE 6:**
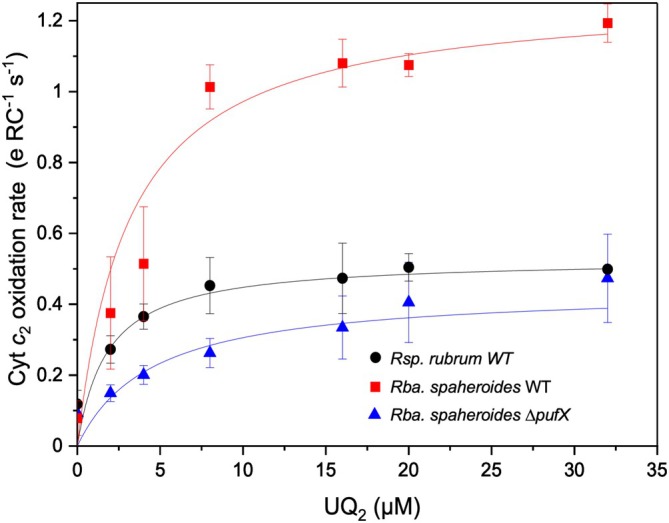
Cytochrome *c*_2_ oxidation rates upon illumination in the presence of various concentrations of ubiquinone‐2 (UQ_2_). See also Figure S10.

Quinones are likely to diffuse more quickly through the membrane than through confined channels within the LH1 ring. To examine the electron transport of RC–LH1 with a closed LH1 ring, we conducted cytochrome *c*_2_ oxidation assays on the RC–LH1 monomer from *Rsp*. *rubrum* in comparison with *Rba. sphaeroides* WT RC–LH1 monomer with an open LH1 ring and *Rba. sphaeroides ΔpufX* RC–LH1 with a closed LH1 ring (Cao et al. 2022). Although the presence of sequestered quinones hinders the precise determination of an apparent Michaelis constant, our results revealed that the maximum quinone/quinol exchange rate for *Rsp*. *rubrum* RC–LH1 monomer (0.52 ± 0.07 e^−^ RC^−1^ s^−1^) was remarkably lower than the open RC–LH1 monomer of *Rba. sphaeroides* WT (1.27 ± 0.08 e^−^ RC^−1^ s^−1^) (Figures 6 and S10). Moreover, it was faster than the closed RC–LH1 monomer of *Rba. sphaeroides ΔpufX* (0.44 ± 0.14 e^−^ RC^−1^ s^−1^), which possesses two carotenoids per LH1 αβ heterodimer (Cao et al. 2022). These results indicate that the LH1 architecture plays a vital role in mediating quinone/quinol trafficking between RC and cytochrome *bc*_1_. A larger opening of the LH1 ring facilitates the rapid diffusion of quinone/quinol molecules across the LH1 barrier. In contrast, the closed RC–LH1 structure of *Rsp*. *rubrum* possesses a small pore between neighboring LH1 αβ‐heterodimers (Figure 3C). While this pore may not be as efficient as the larger opening, it is presumed that it could still enable quinone diffusion through the encircled LH1 ring by the structural flexibility and dynamic “breathing” motion of the complex (Gall et al. 1997; Comayras et al. 2005; Aird et al. 2007; Yu et al. 2018; Qian et al. 2021a; Swainsbury et al. 2021; Tani et al. 2021). Interestingly, the relatively comparable growth of *Rsp*. *rubrum* and *Rba. sphaeroides* suggests that there are other regulatory mechanisms in purple photosynthetic bacteria, such as modulating the abundance and organization of RC–LH1 in ICMs, as well as the morphology and surface area of ICMs (Figure S2). Modulating the abundance and organization of RC–LH1 in ICMs and the morphology and surface area of ICMs in *Rsp*. *rubrum* expands the light‐absorbing capacity, thereby improving its photosynthetic efficiency. This enhancement can increase energy production, compensating for the slower electron transport compared to *Rba. sphaeroides*, ultimately leading to similar growth rates. The presence of additional carotenoids in the LH1 ring of *Rba. sphaeroides ΔpufX*, while potentially enhancing light capture and photoprotection, provides spatial hindrance to restrict quinone/quinol exchange through the pores between adjacent LH1 subunits, thereby causing loss of capability to perform photoheterotrophic growth (Cao et al. 2022).

In summary, the unique features of the RC–LH1 supercomplex from *Rsp*. *rubrum* provide a model system for studying the structure and function of photosynthetic RC–LH1 core complexes. Our cryo‐EM results for *Rsp*. *rubrum* RC–LH1 provide structural insights into the protein–protein and protein‐pigment interaction networks in RC–LH1, which are essential for light‐energy conversion and electron transport. Furthermore, TA spectra and cytochrome *c*_2_ oxidation assays revealed slower LH1 → RC EET rates and quinone diffusion rates of *Rsp*. *rubrum* RC–LH1 with a relatively closed LH1 ring compared to *Rba. sphaeroides* RC–LH1 with a large opening in the LH1 ring. Our integrated approach, which combines structural analysis and spectroscopic investigations, provides an effective method for elucidating the structure and function of natural RC–LH1 core complexes across different species. This approach can also be extended to investigate other photosynthetic supramolecular assemblies from cyanobacteria, algae, and plants, as well as to engineer and characterize artificial photosynthetic biosystems.

## Author Contributions

L.‐N.L. and Y.‐Z.Z. conceived of the study. B.C., P.W., A.M.G., Y.‐Z.Z., and L.‐N.L. designed experiments. B.C., Z.L., P.W., Y.Z., C.W., and A.M.G. performed experiments. B.C., Y.Z., and C.W. purified the protein samples and performed optical spectral analyses. C.W. collected and processed the cryo‐EM data. Z.L. and P.W. assembled and generated the structural model and performed the structural analysis. B.C., Y.Z., and A.M.G. performed TA spectral analysis and cytochrome *c*_2_ oxidation assays. B.C., Z.L., P.W., A.M.G., and L.‐N.L. wrote the manuscript, and all authors contributed to the discussion and improvement of the manuscript.

## Supporting information


**Data S1.** Supporting Information.

## Data Availability

The cryo‐EM density map of *Rsp. rubrum* RC–LH1 monomer was deposited in the Electron Microscopy Data Bank (EMDB, www.ebi.ac.uk/pdbe/emdb/) under the accession code 62025. Atomic coordinates of *Rsp. rubrum* RC–LH1 monomer were deposited in the Protein Data Bank (PDB, www.rcsb.org) with the accession code 9K3Q.
