# Supplementary material for: Characterization of the Structure and Function of the Photosynthetic RC–LH1 Core Supercomplex From Rhodospirillum rubrum
Source: Physiol Plant. 2025 May 19;177(3):e70275. doi: 10.1111/ppl.70275 (PMC12086609; doi:10.1111/ppl.70275)
Supplement: Supplementary file 1 — Data S1. Supporting Information. [file PPL-177-e70275-s001.pdf]

Supplementary Information

Characterization of the structure and function of the photosynthetic RC–LH1 core supercomplex from *Rhodospirillum rubrum*

Bern Christianson[1#], Zekun Liu[2#], Yingyue Zhang[1], Chen Wang[2], Adrian M. Gardner[3], Yu-Zhong Zhang[2,4], Peng Wang[2*], Lu-Ning Liu[1,2*]

[1] Institute of Systems, Molecular and Integrative Biology, University of Liverpool, Liverpool L69 7ZB, United Kingdom

[2] MOE Key Laboratory of Evolution and Marine Biodiversity, Frontiers Science Center for Deep Ocean Multispheres and Earth System & College of Marine Life Sciences, Ocean University of China, Qingdao 266003, China

[3] Department of Chemistry, Stephenson Institute of Renewable Energy, and Early Career Laser Laboratory, University of Liverpool, Liverpool L69 7ZF, United Kingdom

[4] Marine Biotechnology Research Center, State Key Laboratory of Microbial Technology, Shandong University, Qingdao 266237, China

[#] These authors contributed equally to this work.

*** Correspondence:**

Peng Wang,

Email: wangpeng3331@ouc.edu.cn;

Lu-Ning Liu.

Email : luning.liu@liverpool.ac.uk

Supplementary Methods

Analysis of TA data

The TA spectral features were assigned based on those observed in the ground-state UV/Vis spectra and those reported in TA spectra of other RC–LH1 complexes or isolated RCs (Šlouf et al. 2012, Šlouf et al. 2013, Thwaites et al. 2023) (Fig. S6, Fig. S1D). At early time frames (1–10 ps), the spectra were primarily dominated by signals from the LH1 chromophores. A characteristic band at 877 nm was assigned to ^{LH1}BChl(Q_y); a negative peak at 895 nm corresponded to the overlapping ground-state bleach (GSB) and stimulated emission (SE) of ^{LH1}BChl(Q_y), whereas a positive peak at 860 nm was attributed to the photoinduced absorption (PIA) of ^{LH1}BChl(Q_y)*. Below 750 nm, a broad PIA with a

narrow negative feature at 590 nm was observed, which was assigned to the GSB of $^{LH1}BChl(Q_x)$. At 50 ps and 500 ps, spectral features associated with the RC chromophores emerged, accompanied by a reduction in intensity of the LH1 bands, indicative of LH1→RC EET.

Several methods are commonly employed to provide kinetic insight into complex datasets generated from time-resolved spectroscopies. These have been reviewed (Ruckebusch et al. 2012, Slavov et al. 2015, Forster et al. 2020, Slavov et al. 2020), and we have recently discussed the analysis of TA spectra of RC–LH1 complexes (Thwaites et al. 2023). In this work, we employ lifetime density analysis (LDA) to examine the RC–LH1 kinetics. LDA is based on the principle that the time-resolved data can be represented by a continuous distribution of single exponential functions, Equation 1, where $\emptyset(\tau)$ is the spectral distribution function. To make Equation 1 readily solvable, the integral needs to be discretized into a quasi-continuous sum of *n* exponential functions (*n* = 500 in this work).

$$\Delta \mathrm{A}(\tau, \lambda) = \int_0^\infty \emptyset(\tau,\ \lambda)\ e^{-\frac{t}{\tau}} \qquad (1)$$

LDA results in a three-dimensional density map, $x(\tau,\lambda)$, termed a “lifetime density map”, shown in Fig. S8B for *Rsp. rubrum*. Comparison of lifetime density maps is complicated owing to the difficulty in accurately representing the magnitude of the pre-exponential factor with contour/colour maps. Instead, we reduce the three-dimensional lifetime density map to a series of two-dimensional plots. Information on the kinetics is obtained through the integration of the modulus of the pre-exponential factor between 750 – 950 nm for each lifetime, (which displays the most intense TA spectra features), which we term lifetime density kinetic trace, LDKT. The wavelength dependent average pre-exponential factor of lifetimes associated with each band observed in the LDKT can be calculated, allowing the spectral change associated with each kinetic process to be plotted in two dimensions, which we denote as lifetime averaged difference spectra, LADS. LDKT are shown in Fig. 5 obtained from LDA of a typical TA spectrum of the RC–LH1 supercomplex of *Rsp. rubrum*. LADS indicate the change that occurs in the TA spectra throughout the distribution of lifetimes included within the average. Owing to this, a positive feature observed within LADS indicates the decay of a positive TA band, or growth of a negative TA band. Similarly, a negative feature indicates the decay of a negative TA band, or the growth of a positive TA band.

Inspection of the LADS corresponding to the feature observed with a peak at ~50 ps in the LDKT of Fig. S8C shows loss of LH1 TA bands [loss of ^{LH1}BChl(Qy) GSB and ^{LH1}BChl(Qy)* SE signals at wavelengths longer than ~890 nm and ^{LH1}BChl(Qy)* PIA at ~860 nm] contemporaneously with the growth of RC features [^{RC}BChl(Qy) derivative line shape band at ~800 nm and ^{RC}BPhe(Qy) GSB at ~760 nm, hence corresponds to LH1→RC EET. The LADS corresponding to the kinetic feature with a peak lifetime of ~4 ps shows only features assigned to loss of LH1 TA bands, loss of ^{LH1}BChl(Qy) GSB and ^{LH1}BChl(Qy)* SE signals at wavelengths longer than ~890 nm and ^{LH1}BChl(Qy)* PIA at ~860 nm,

consistent with exciton-exciton annihilation and ^{LH1}BChl(Qy)* → ^{LH1}BChl(Qy) relaxation. It is important to avoid excessive analysis of the weaker features observed within the LDKT, which may arise from residual noise in the analysis as well as the blurring of kinetic processes within the broad baseline, hence we do not analyse the LADS corresponding to other features observed in the LDKT spectrum of Fig. S8C (Thwaites et al. 2023, Wang et al. 2024).

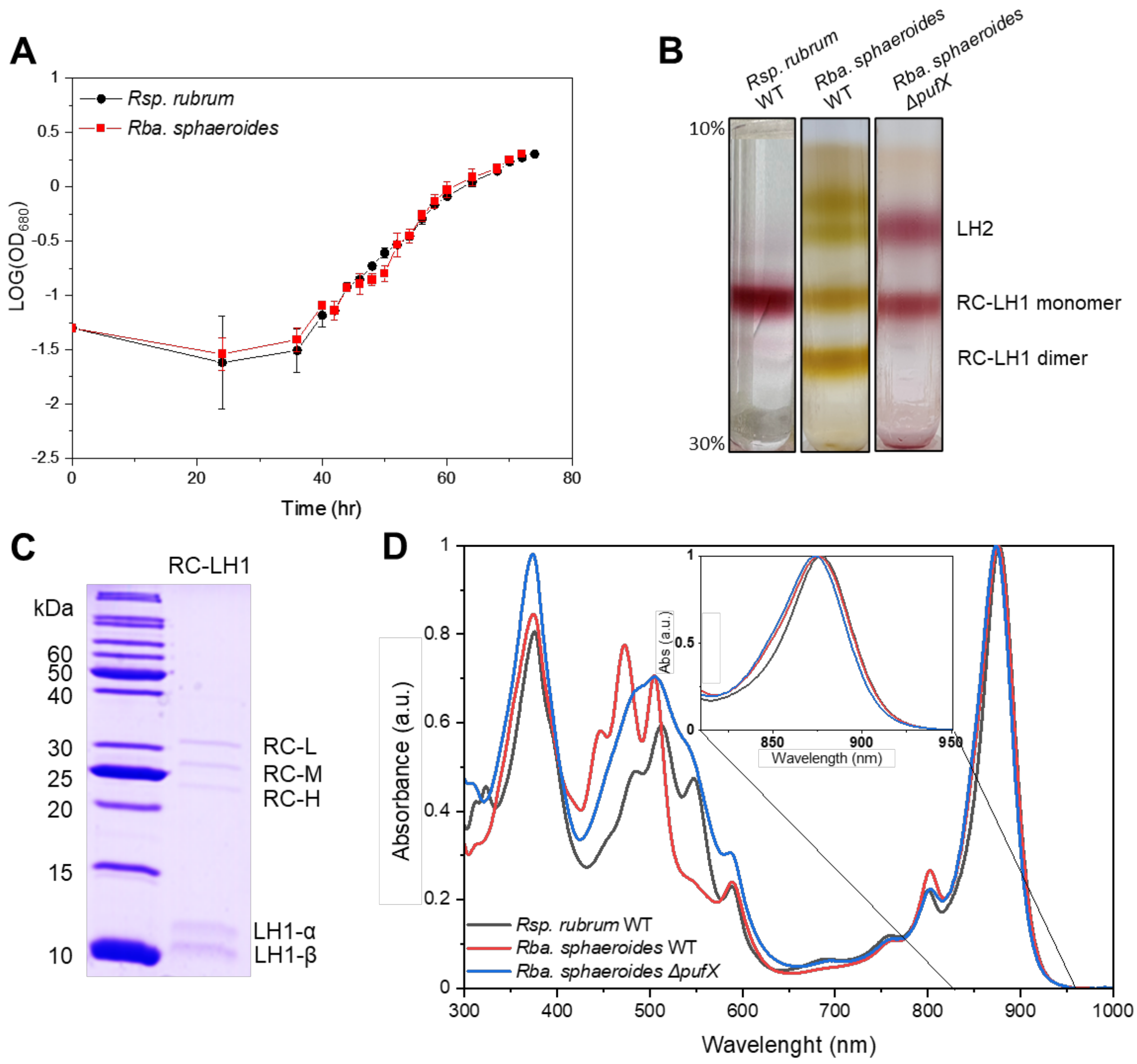

Fig. S1. Cell growth and RC–LH1 purification and characterization. (A) Phototrophic growth of the wild-type (WT) cells of *Rsp. rubrum* and *Rba. sphaeroides*. (B) Sucrose gradient ultracentrifugation of photosynthetic membrane complexes from *Rsp. rubrum* WT (left), *Rba. sphaeroides* WT (middle), and *Rba. sphaeroides ΔpufX* (right). (C) SDS-PAGE of purified RC–LH1 complexes from *Rsp. rubrum.* (D) Room-temperature UV-vis absorbance spectra (astronomical unit: a.u.) of purified RC–LH1 monomers from *Rsp. rubrum* WT, *Rba. sphaeroides* WT, and *Rba. sphaeroides ΔpufX.* Inset shows a zoom of the ^{LH1}BChl(Qy) absorption peak.

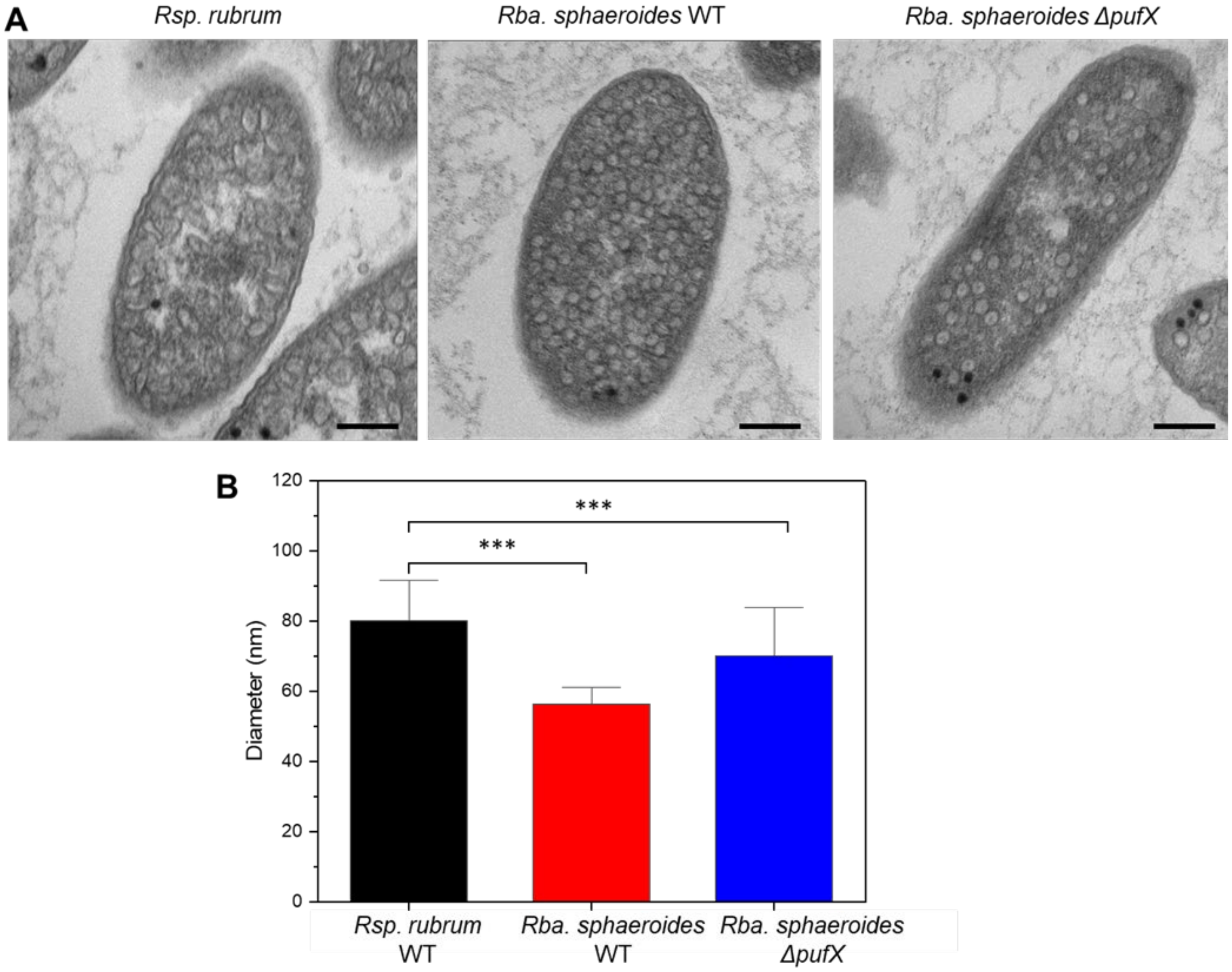

Fig. S2. Analysis of ICMs in purple photosynthetic bacteria. (A) Thin-section transmission electron microscopic images of the cells of *Rsp. rubrum* (left), *Rba. sphaeroides* WT (middle) and *Rba. sphaeroides* Δ*pufX* (right). Scale bar: 200 nm. (B) Apparent diameters of ICM vesicles measured from (A). *** $p = 1.8 \times 10^{-22}$, *Rsp. rubrum* WT vs. *Rba. sphaeroides* WT; *** $p = 3.8 \times 10^{-5}$, *Rsp. rubrum* WT vs. *Rba. sphaeroides* Δ*pufX*. n = 100 ICM vesicles per sample. Welch's t-test was used for statistical analysis.

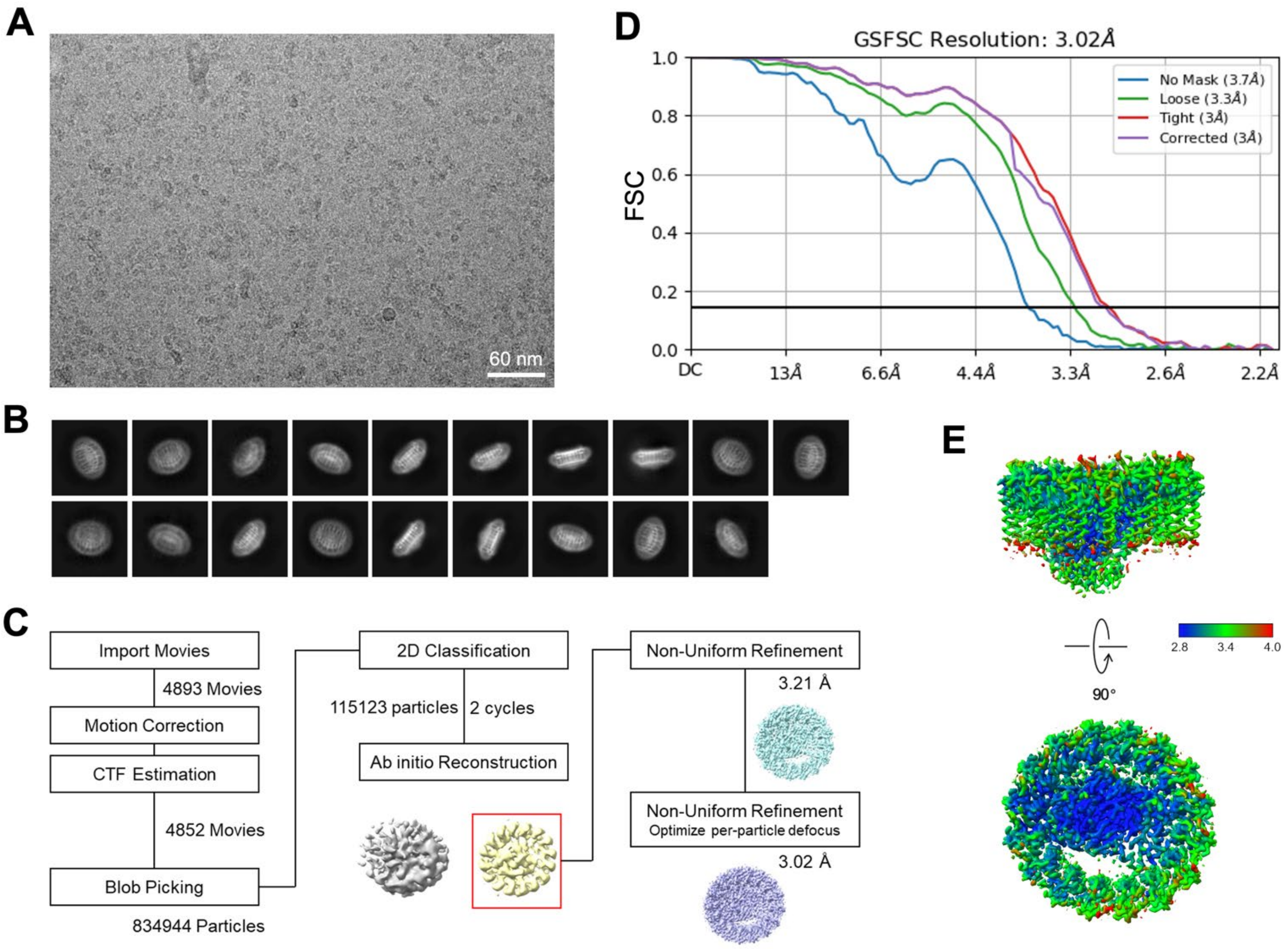

Fig. S3. Cryo-EM data process of *Rsp. rubrum* RC–LH1. (A) Motion-corrected example of a cryo-EM captured movie. (B) Representative reference-free 2D class averages. (C) Overview of cryo-EM data processing. Selected 3D class that went into further processing is marked with a red rectangle. (D) Fourier Shell Correlation (FSC) curves generated by cryoSPARC. Global resolution values were calculated according to the gold-standard FSC = 0.143. (E) Local resolution of the cryo-EM map.

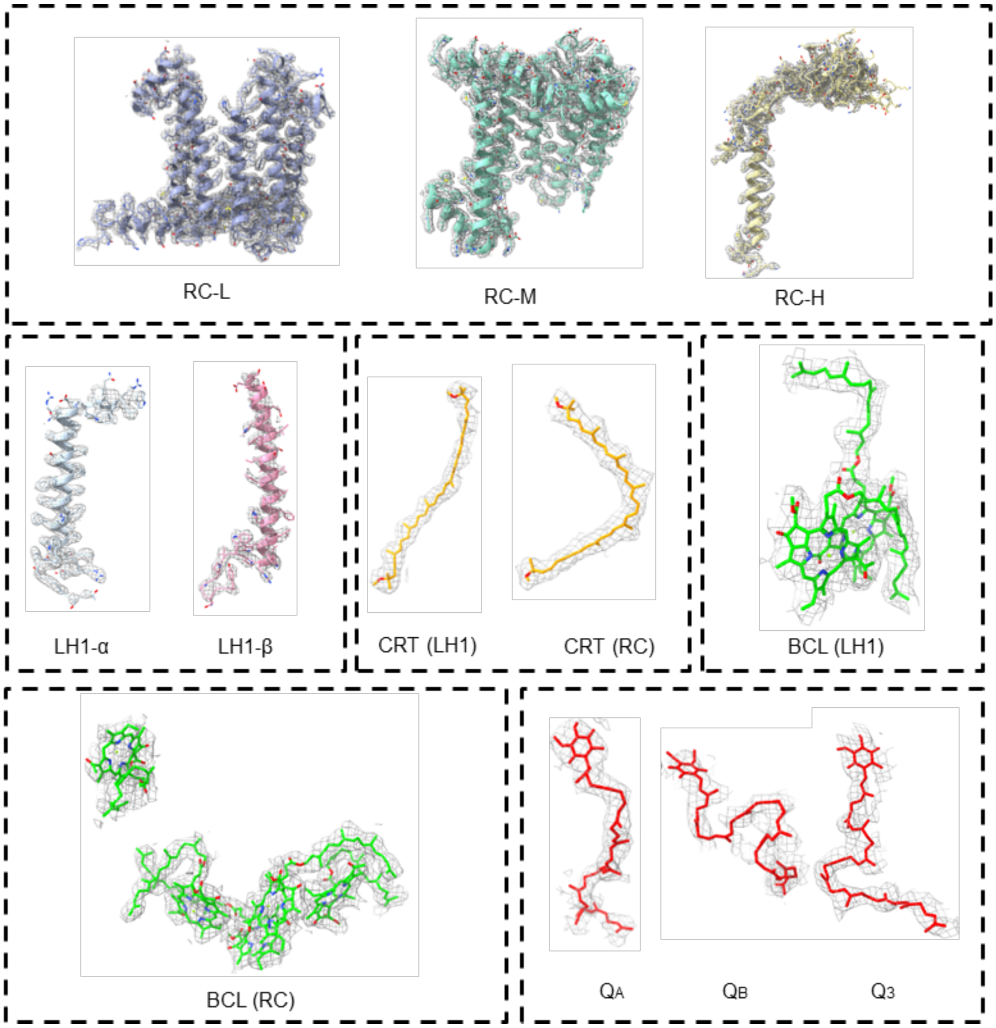

Fig. S4. Cryo-EM map densities and structural models of protein peptides and cofactors in the *Rsp. rubum* RC–LH1 monomer.

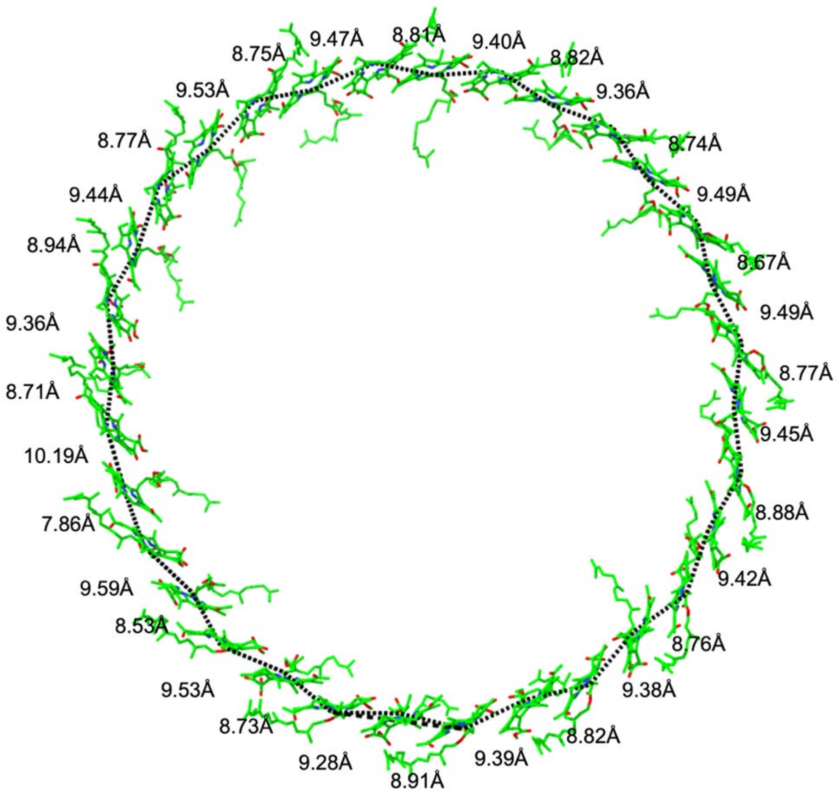

Fig. S5. Intra-and inter-subunit Mg-Mg distances between the BChls within LH1 from *Rsp. rubrum*.

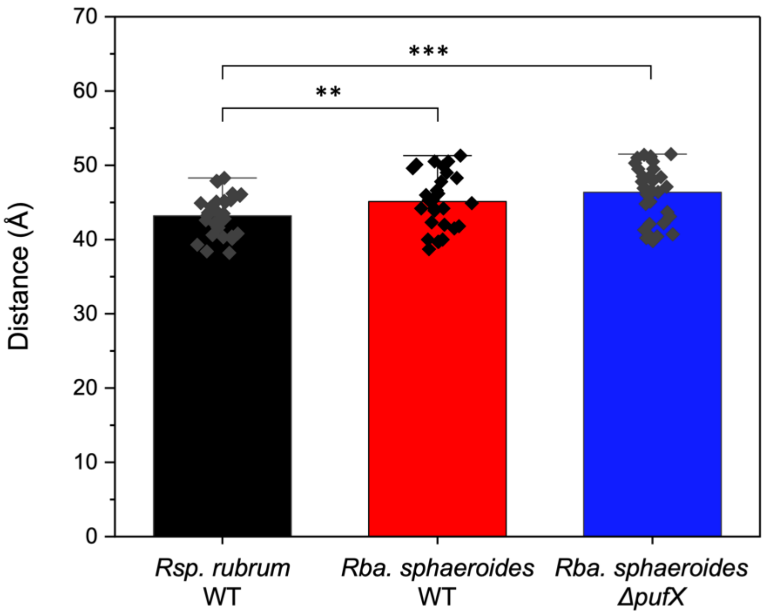

Fig. S6. ^{LH1}BChl–^{RC}P distances between RC–LH1 monomers. ** p =0.0091, *Rsp. rubrum* WT vs. *Rba. sphaeroides* WT; *** p =3.8 x10^{-5}, *Rsp. rubrum* WT vs. *Rba. sphaeroides* Δ*pufX* (Welch's t-test was used for statistical comparison).

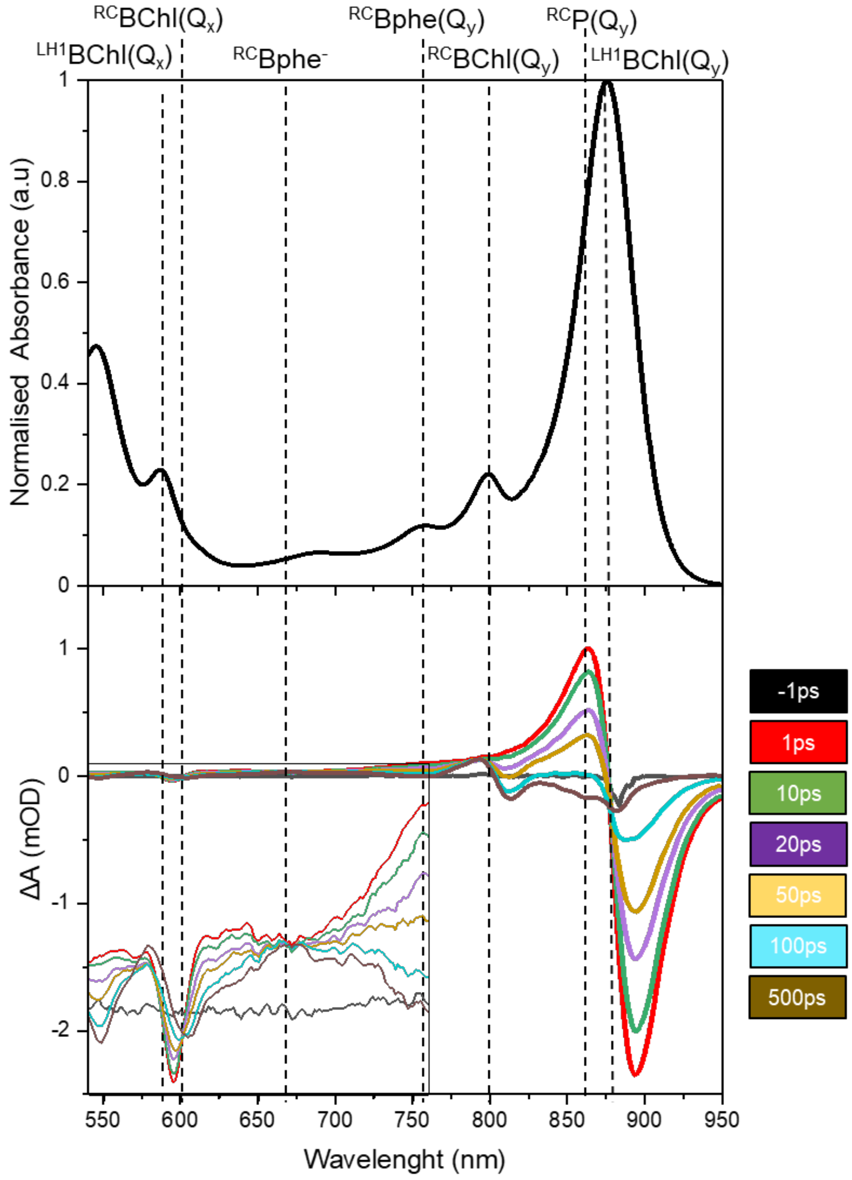

Fig. S7. UV/vis (top) and transient absorption (TA, bottom) spectra of RC–LH1 from *Rsp rubrum*. TA spectra were recorded at selected timepoints. Inset, magnified TA spectra over the 550-750 nm spectral region.

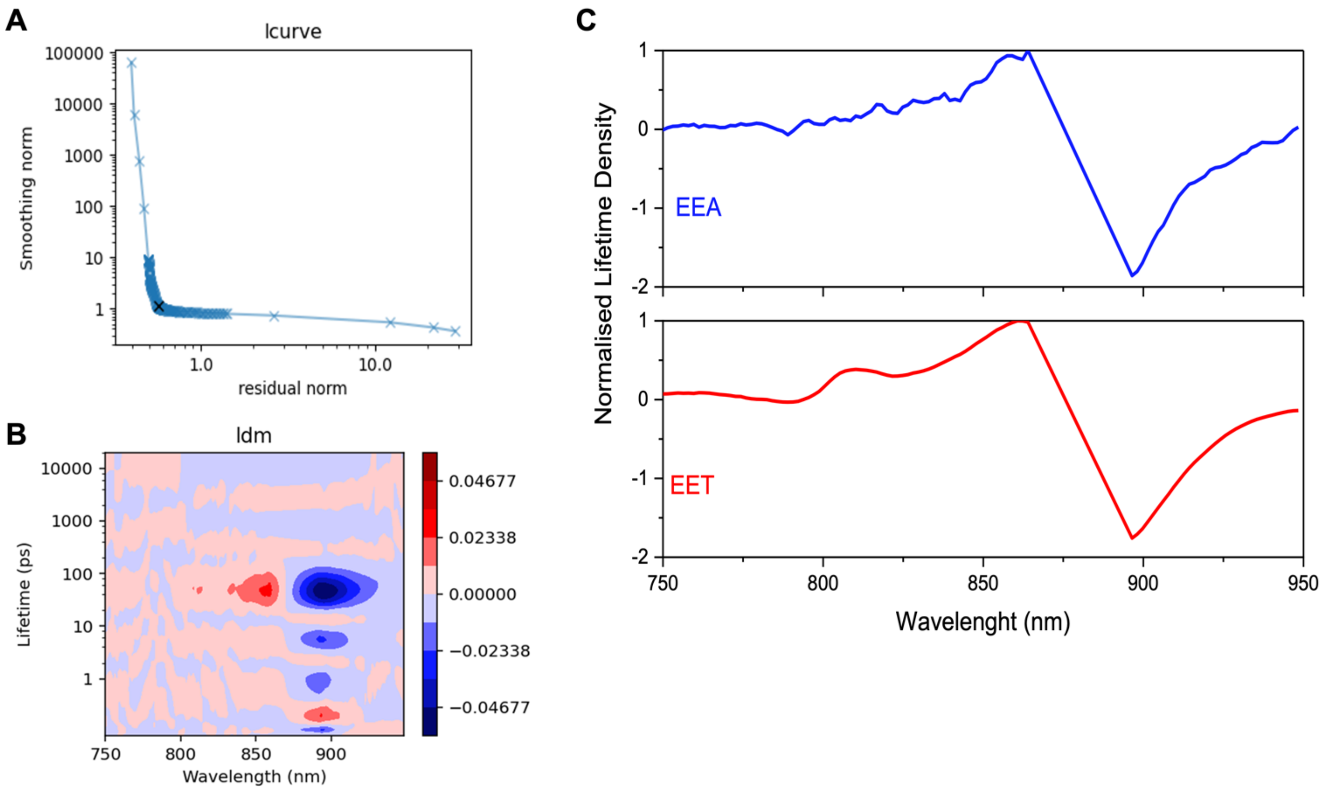

Fig. S8. TA spectral analysis of *Rsp. rubrum* RC–LH1. (A) L-curves obtained from the lifetime density analysis (LDA) fitting process for *Rsp. rubrum* RC–LH1. (B) Lifetime density map generated from LDA of TA spectra for *Rsp. rubrum* RC–LH1 complexes. (C) The lifetime averaged difference spectra showing the wavelength dependent average pre-exponential factor of lifetimes of certain timescale. (Top) LADS associated with the process at ~4 ps show the decay of LH1 chromophore TA bands, consistent with exciton-exciton annihilation (EEA), and relaxation of excited Qy bacteriochlorophyll (^{LH1}BChl (Qy)*→^{LH1}BChl (Qy)). (Bottom) LADS associated with the process at ~50 ps show energy transfer from bacteriochlorophyll in LH1 (^{LH1}BChls) to bacteriochlorophyll special pair in the reaction centre (^{RC}P) through EET.

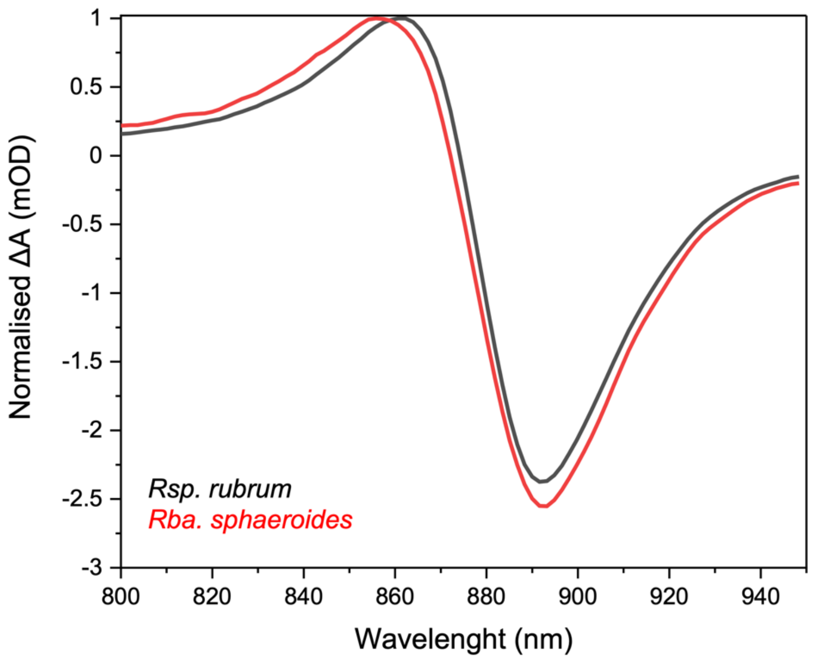

Fig. S9. TA spectra comparison at 2 ps of *Rsp. rubrum* WT *and Rba. sphaeroides* WT.

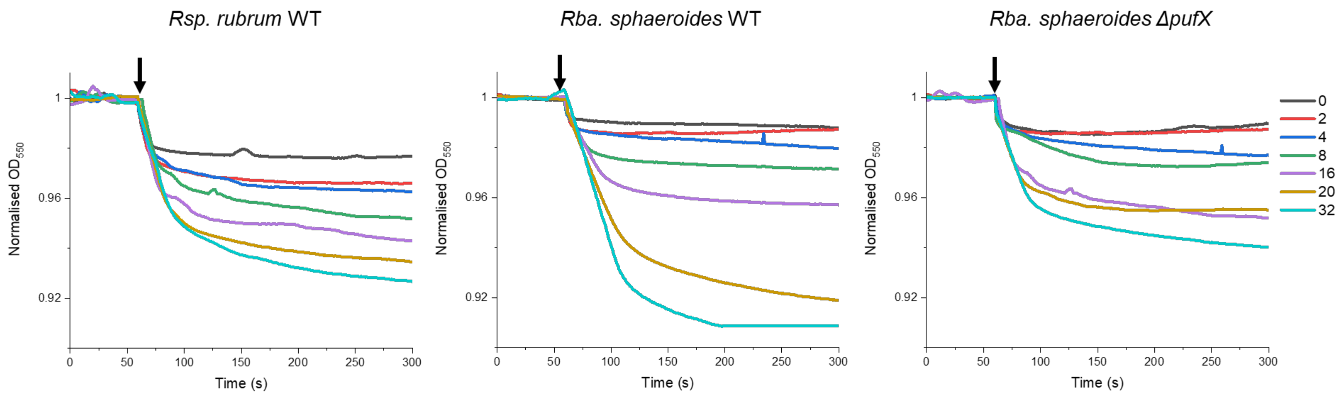

Fig. S10. Absorbance changes at 550 nm upon illumination of *Rsp. rubrum* RC–LH1 monomer in comparison with RC–LH1 monomers from *Rba. sphaeroides* WT and Δ*pufX*. Assays were carried out with 15 nM of RC–LH1 monomers. The RC–LH1 complexes were incubated with 30 μM reduced cytochrome c_2 and various concentrations of UQ_2 (μM), as indicated, at 4°C overnight. To account for variability of starting OD_{550} between samples, we normalized the data to the average OD_{550} during the 1-minute steady-state period before illumination. Light was turned off for the initial 1-minute to ensure stable reduced cytochrome c_2 condition. Black arrows indicate the beginning of the illumination period and the start of cytochrome c_2 oxidation process. Measurement was conducted for 5 minutes to ensure the cytochrome c_2 oxidation reached steady-state condition during illumination. Cytochrome c_2 oxidation rates dependent on UQ_2 concentrations were determined by fitting the linear initial rate before the equilibrium phase, from 1 s to 10 s. Note that the cytochrome turnover rates appeared to be dependent on the quinone concentration, consistent with previous observations (Osvath and Maroti 1997).

Table S1. Cryo-EM data collection, refinement and validation statistics.

	Rsp. rubrum RC–LH1 **(EMD-62025; PDB: 9K3Q)**
Data collection and processing	
Magnification (nominal)	81,000
Voltage (kV)	300
Detector	Gatan K3
Electron exposure ($e^-/Å^2$)	50.0
Defocus range (μm)	-0.8~-1.8
Pixel size (Å)	0.53
Symmetry imposed	C1
Initial particle images (no.)	834,944
Final particle images (no.)	97,584
FSC threshold	0.143
Map resolution (Å)	3.02
Refinement	
Initial model used (PDB code)	7OY8
Model resolution (Å)	2.50
FSC threshold	0.143
Model composition	
Non-hydrogen atoms	21,510
Protein residues	2,212
*B*factor ($Å^2$)	
Protein	67.26
R.m.s deviations	
Bond lengths (Å)	0.011
Bond angles (°)	1.560
Validation	
MolProbity score	1.62
Clashscore	8.11
Poor rotamers (%)	0.66
Ramanchandran plot	
Favored (%)	96.97
Allowed (%)	3.03
Disallowed (%)	0

Table S2. Interactions within the RC−LH1 assembly.

Atom1	Atom2	Distance (Å)
/2 SER34 OG	/M SER 81 OG	1.766
/E ASN 39 ND2	/H ILE 6 O	2.913
/F GLN 12 NE2	/H VAL 59 O	3.693
/F SER 34 OG	/H THR 7 OG1	2.693
/H THR 7 OG1	/F SER 34 OG	2.693
/H SER 58 OG	/G GLN 12 OE1	3.555
/L TRP 52 NE1	/N SER 34 OG	2.922
/L TRP 60 NE1	/J SER 34 OG	2.610
/M ARG 134 NH2	/8 GLN 12 OE1	2.904
/Z SER 34 OG	/L PRO 271 O	2.813
/L GLY 19 N	/A GLN 12 OE1	2.907

Table S3. Interactions within the LH1 assembly.

Atom1	Atom2	Distance (Å)
/F TRP 5 NE1	/O HIS 21 ND1	3.055
/O ARG 46 NH1	/F ARG 37 O	2.332
/O ARG 46 NH1	/F ALA 44 O	3.418
/O ARG 46 NH2	/F ALA 44 O	2.821
/F TRP 48 NE1	/O 07D 1001 OBB	2.704
/O TRP 40 NE1	/O 07D 102 OBB	2.726
/O PRO 47 O	/F ARG 37 NE	3.255
/O PRO 47 O	/F ARG 37 NH2	3.188
/6 ARG 11 NH1	/8 LEU 7 O	3.348
/6 ARG 11 NH2	/8 LEU 7 O	2.908
/8 GLU 36 N	/6 GLU 42 OE1	3.224
/6 SER 45 OG	/9 PRO 52 O	3.045
/6 THR 46 N	/9 PRO 52 O	2.831
/6 THR 46 OG1	/9 PRO 52 O	3.080
/9 TYR 55 OH	/6 TRP 40 O	2.529
/7 TRP 45 NE1	/9 PRO 50 O	2.780
/7 ARG 46 NH2	/9 GLY 51 O	2.974
/4 THR 46 N	/7 PRO 52 O	2.824
/7 TYR 55 OH	/4 TRP 40 O	2.436
/5 TRP 45 NE1	/7 PRO 50 O	2.693
/5 ARG 46 NH2	/7 GLY 51 O	2.924

Table S4. BChl *a*-BChl *a* distance to the neighboring LH1 (^{LH1}BChl–^{LH1}BChl distances).

Atom1	Atom2	Distance (Å)
/5 07D	/4 07D	9.38
/7 07D	/4 07D	8.76
/7 07D	/6 07D	9.42
/6 07D	/9 07D	8.88
/8 07D	/9 07D	9.45
/8 07D	/I 07D	8.77
/D 07D	/I 07D	9.49
/D 07D	/K 07D	8.67
/E 07D	/K 07D	9.49
/E 07D	/O 07D	8.74
/F 07D	/O 07D	9.36
/F 07D	/Q 07D	8.82
/G 07D	/Q 07D	9.40
/G 07D	/S 07D	8.81
/J 07D	/S 07D	9.47
/J 07D	/U 07D	8.75
/N 07D	/U 07D	9.53
/N 07D	/W 07D	8.77
/A 07D	/W 07D	9.44
/A 07D	/Y 07D	8.94
/R 07D	/Y 07D	9.36
/R 07D	/d 07D	8.71
/T 07D	/d 07D	10.19
/T 07D	/m 07D	7.86
/V 07D	/m 07D	9.59
/V 07D	/n 07D	8.54
/X 07D	/n 07D	9.53
/X 07D	/1 07D	8.73
/Z 07D	/1 07D	9.39
/Z 07D	/3 07D	8.91
/2 07D	/3 07D	9.39
/2 07D	/5 07D	8.82

Table S5. Distances between the BChls a in LH1 to the special pair of BChls *a* in the RC (^{LH1}BChl–^{RC}P distances).

Atom1	Atom2	Distance (Å)
/3 07D	/L 07D	48.36
/2 07D	/M 07D	47.62
/5 07D	/M 07D	48.43
/4 07D	/M 07D	45.00
/7 07D	/M 07D	45.66
/6 07D	/M 07D	42.46
/9 07D	/M 07D	43.03
/8 07D	/M 07D	39.60
/I 07D	/M 07D	40.02
/K 07D	/M 07D	39.52
/D 07D	/M 07D	37.73
/Z 07D	/L 07D	45.30
/1 07D	/L 07D	45.88
/X 07D	/L 07D	42.43
/n 07D	/L 07D	43.30
/V 07D	/L 07D	40.18
/m 07D	/L 07D	41.74
/T 07D	/L 07D	40.99
/d 07D	/L 07D	41.63
/R 07D	/L 07D	40.64
/Y 07D	/L 07D	43.66
/A 07D	/L 07D	42.56
/W 07D	/L 07D	45.41
/N 07D	/L 07D	44.02
/U 07D	/L 07D	46.32
/J 07D	/H 07D	44.47
/S 07D	/M 07D	44.84
/G 07D	/M 07D	41.46
/Q 07D	/M 07D	42.22
/F 07D	/M 07D	39.15
/O 07D	/M 07D	40.15
/E 07D	/M 07D	37.59

Table S6. Intra-and inter-subunit Mg-Mg distances between the BChls within RC–LH1 complexes from various purple photosynthetic bacteria.

RC–LH1	LH1–LH1 distance (Å)	
	Intra	Inter
Rsp. rubrum WT	9.5 ± 0.2	8.7 ± 0.2
Tch. tepidum WT	8.9 ± 0.1	8.7 ± 0.1
Trv. strain 970	9.0 ± 0.1	8.8 ± 0.1
Rps. palustris WT	9.6 ± 0.1	8.3 ± 0.2
Rfl. castenholzii WT	9.5 ± 0.3	9.3 ± 0.2
Rba. blasticus WT	9.9 ± 0.2	8.2 ± 0.4
Rba. sphaeroides WT	9.4 ± 0.1	8.6 ± 0.1

Supplementary References:

Forster M, Cheung DW, Gardner AM, Cowan AJ (2020) Potential and pitfalls: On the use of transient absorption spectroscopy for in situ and operando studies of photoelectrodes. The Journal of Chemical Physics 153(15)

Osvath S, Maroti P (1997) Coupling of cytochrome and quinone turnovers in the photocycle of reaction centers from the photosynthetic bacterium Rhodobacter sphaeroides. Biophys J 73(2): 972-982

Ruckebusch C, Sliwa M, Pernot Pd, De Juan A, Tauler R (2012) Comprehensive data analysis of femtosecond transient absorption spectra: A review. Journal of Photochemistry and Photobiology C: Photochemistry Reviews 13(1): 1-27

Slavov C, Hartmann H, Wachtveitl J (2015) Implementation and evaluation of data analysis strategies for time-resolved optical spectroscopy. Analytical chemistry 87(4): 2328-2336

Slavov C, Fischer T, Barnoy A, Shin H, Rao AG, Wiebeler C, Zeng X, Sun Y, Xu Q, Gutt A (2020) The interplay between chromophore and protein determines the extended excited state dynamics in a single-domain phytochrome. Proceedings of the National Academy of Sciences 117(28): 16356-16362

Šlouf V, Chábera P, Olsen JD, Martin EC, Qian P, Hunter CN, Polívka T (2012) Photoprotection in a purple phototrophic bacterium mediated by oxygen-dependent alteration of carotenoid excited-state properties. Proceedings of the National Academy of Sciences 109(22): 8570-8575

Šlouf Vc, Fuciman M, Dulebo A, Kaftan D, Koblížek M, Frank HA, Polívka Ts (2013) Carotenoid charge transfer states and their role in energy transfer processes in LH1–RC complexes from aerobic anoxygenic phototrophs. The Journal of Physical Chemistry B 117(38): 10987-10999

Thwaites O, Christianson BM, Cowan AJ, Jäckel F, Liu L-N, Gardner AM (2023) Unravelling the roles of integral polypeptides in excitation energy transfer of photosynthetic RC-LH1 supercomplexes. The Journal of Physical Chemistry B 127(33): 7283-7290

Wang P, Christianson BM, Ugurlar D, Mao R, Zhang Y, Liu Z-K, Zhang Y-Y, Gardner AM, Gao J, Zhang Y-Z (2024) Architectures of photosynthetic RC-LH1 supercomplexes from Rhodobacter blasticus. Science advances 10(41): eadp6678
